# Supplementary material for: Monolayer Kagome metals AV3Sb5
Source: Nat Commun. 2023 Feb 3;14:591. doi: 10.1038/s41467-023-36341-2 (PMC9898313; doi:10.1038/s41467-023-36341-2)
Supplement: Supplementary file 1 — Supplementary Information [file 41467_2023_36341_MOESM1_ESM.pdf]

Supplementary Information for "Monolayer Kagome Metals AV_3Sb_5 "

Sun-Woo Kim[1,2,3,*], Hanbit Oh[2,*], Eun-Gook Moon[2,†], Youngkuk Kim[1,‡]

[1] *Department of Physics, Sungkyunkwan University, Suwon 16419, Republic of Korea*

[2] *Department of Physics, KAIST, Daejeon, 34126, Republic of Korea*

[3] *Department of Materials Science and Metallurgy, University of Cambridge, 27 Charles Babbage Road, Cambridge CB3 0FS, United Kingdom*

Contents

Supplementary Note 1. Energetics of various monolayer structures 3

Supplementary Note 2. Rb and Cs systems 5

2.1. Band structures and VHS of the pristine phase 5

2.2. Phonon softening of the pristine phase 6

2.3. Phonon spectra of the CDW phase 7

Supplementary Note 3. Analysis on the VHS points 9

3.1. Sublattice character of VHS 9

3.2. Rearrangement of VHS 10

Supplementary Note 4. Sublattice resolved susceptibility 13

Supplementary Note 5. Detailed phonon analysis 15

5.1. Leading CDW instability of the pristine phase 15

5.2. Correction of artificial negative phonon modes of the ISD-1 phase 17

Supplementary Note 6. Details on various CDW orders 18

6.1. Anisotropic 2×2 $3Q$ CDW order 18

6.2. Band structures of ISD-1 and SD-2 CDWs 18

6.3. TRSB CDW: Comparison with the D_{6h} bulk 19

Supplementary Note 7. Details on tight-binding Hamiltonian 22

7.1. Notation 22

7.2. Tight-binding Hamiltonian in 2×2 unit cell 22
7.3. Justification of the tight-binding model 24

Supplementary Note 8. Details on mean-field analysis 26
8.1. Model and strategy 26
8.2. Charge density wave (CDW) 27
8.3. Superconductivity (SC) 30
8.4. Details of variational method 33
8.5. Comment on the attractive interaction 33

Supplementary Note 9. Details on mean-field phase diagram 35

Supplementary Note 10. Estimation of U and V values and their tunability 39

Supplementary Note 11. Comments on Incommensurate CDW 41

Supplementary Note 12. Engineering of VHS 47

Supplementary References 48

Supplementary Note 1. Energetics of various monolayer structures

In this section, we provide details about the constraint of stoichiometry on the monolayer structure and about the energetics of the monolayer structure satisfying the stoichiometry of AV_3Sb_5. We first present the effect of stoichiometry on the stability of monolayer structures by comparing the cohesive energy between monolayer structures with different stoichiometry. The cohesive energy per atom E_{coh} is given by

$$E_{coh} = [n_A E_A + n_{\mathrm{V}} E_{\mathrm{V}} + n_{\mathrm{Sb}} E_{\mathrm{Sb}} - E_{tot}]/(n_A + n_{\mathrm{V}} + n_{\mathrm{Sb}}), \quad (1)$$

where E_A, E_{V}, E_{Sb}, and E_{tot} are the total energies of a single A atom, a single V atom, a single Sb atom, and monolayer, respectively. The cohesive energy E_{coh} is calculated as 3.8 (3.6) eV for the monolayer structures having the stoichiometry of AV_3Sb_5 ($A_2V_3Sb_5$), which implies that the monolayer structure with the stoichiometry $A_2V_3Sb_5$ is less likely realized in the experiment. This is in line with the experimental observations [1, 2] that show the A-terminated surface is unstable for exfoliated samples because A atoms are prone to form a cluster. Thus, we only consider the monolayer structure satisfying the stoichiometry of

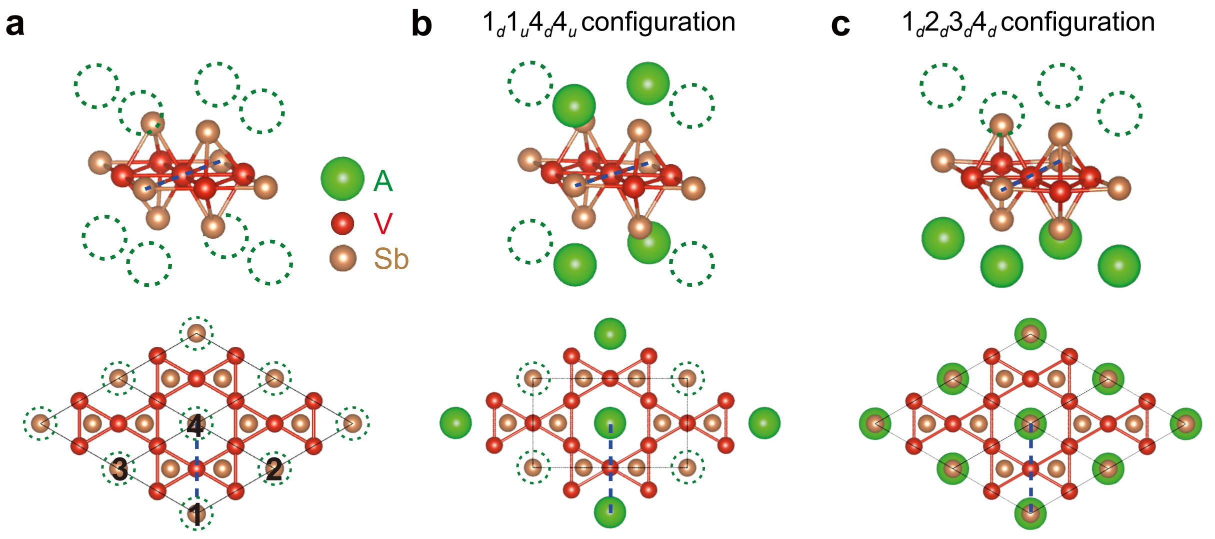

FIG. S1: **Various monolayer structures satisfying the stoichiometry of AV_3Sb_5. a** Possible atomic sites (green dashed open circles; labeled as 1 to 4 with up and down positions relative to V-Sb triple layers) of alkali metal atoms A in the monolayer structure. Black lines indicate the 1×1 unit cell. Dashed blue lines are drawn to show the direction in the geometries. **b,c** Two exemplary configurations $1_d1_u4_d4_u$ and $1_d2_d3_d4_d$. The $1_d1_u4_d4_u$ configuration is the lowest energy structure of the monolayer AV_3Sb_5 which is studied in the main text.

$A\mathrm{V}_3\mathrm{Sb}_5$.

Next, keeping the stoichiometry of $A\mathrm{V}_3\mathrm{Sb}_5$, we obtain energetics of various monolayer structures by considering all the possible configurations of the alkali atoms (Fig. S1 and Table S1). There are six symmetrically distinct monolayer structures depending on the configurations of the alkali atoms: $1_d1_u4_d4_u$, $1_d1_u2_d3_d$, $1_d1_u2_d3_u$, $1_d2_d3_d4_d$, $1_d2_d3_d4_u$, and $1_d2_u3_d4_u$ where 1_d refers the position of a alkali atom located at site 1 (see site indices in Fig. S1**a**) and below the V-Sb triple layers ("down" position; d). The configurations of the alkali atoms determine the lattice type and symmetry of monolayer structures (see Table S1). We find that $1_d1_u4_d4_u$ structure having rectangular lattice with $\mathcal{T}_{\sqrt{3}\times 1}$ translational and D_{2h} point group symmetries (Fig. S1**b**) is the most energetically favorable for all three alkali atoms. Note that the rectangular lattice is commonly observed in the surface of exfoliated bulk [3–5], which supports our calculation results.

TABLE S1: **Lattice type, symmetry, and energetics for various pristine monolayer structures satisfying the chemical stoichiometry of $A\mathrm{V}_3\mathrm{Sb}_5$.** Six symmetrically distinct configurations of A atoms are given. The configurations are labelled by the site index i ($i = 1, 2, 3, 4$) and height index u or d (up and down positions relative to the V-Sb triple layers), as shown in Fig. S1. For energetics, zero energy is set to the lowest total energy in each column.

Configuration	Lattice	Symmetry	Energetics (Unit: meV/f.u.)		
			K	Rb	Cs
$1_d1_u4_d4_u$	Rectangular	$\mathcal{T}_{\sqrt{3}\times 1}$ D_{2h}	0.0	0.0	0.0
$1_d1_u2_d3_d$	Triangular	$\mathcal{T}_{2\times 2}$ C_{2v}	29.4	39.3	49.2
$1_d1_u2_d3_u$	Triangular	$\mathcal{T}_{2\times 2}$ D_2	0.3	0.7	1.1
$1_d2_d3_d4_d$	Triangular	$\mathcal{T}_{1\times 1}$ C_{6v}	125.9	166.4	206.5
$1_d2_d3_d4_u$	Triangular	$\mathcal{T}_{2\times 2}$ C_{2v}	30.9	41.1	51.1
$1_d2_u3_d4_u$	Triangular	$\mathcal{T}_{2\times 2}$ S_2	1.7	2.7	3.2

Supplementary Note 2. Rb and Cs systems

2.1. Band structures and VHS of the pristine phase

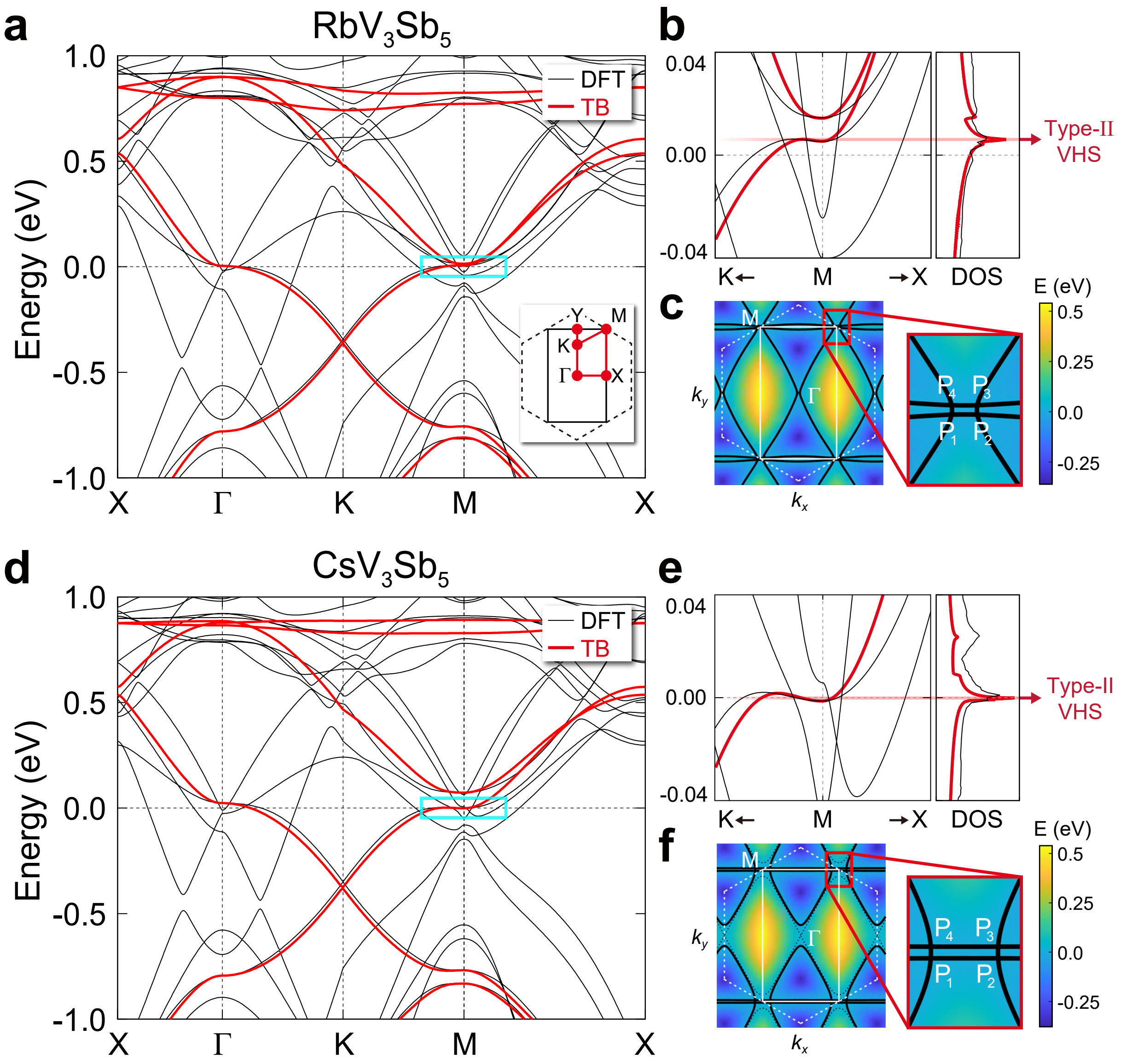

FIG. S2: **Electronic structures and type-II VHS points in the RbV_3Sb_5 and CsV_3Sb_5 monolayer.** **a** DFT and TB band structures of the RbV_3Sb_5 monolayer. The black and red color schemes are used to indicate the DFT and TB bands. Solid (dashed) line in inset of **a** depicts the BZ of the $\sqrt{3} \times 1$ (1×1) unit cell. **b** Magnified view of the boxed region in **a**. The density of states (DOS) is also shown where the energy of type-II VHSs is indicated by the red arrow. **c** Energy contours containing saddle points of the TB bands. White dashed (solid) lines show the pristine (reduced) BZ. The black solid and dashed contour curves correspond to $E = 6$ and 4 meV, respectively. **d-f** Same data set as **a-c** but for the CsV_3Sb_5 monolayer. In **f**, the black solid and dashed contour curves correspond to $E = 0$ and 24 meV, respectively.

Figure S2 shows the band structures and type-II VHSs of the RbV_3Sb_5 and CsV_3Sb_5 monolayer. We find that the TB band structures well reproduce the VHS bands of the DFT results for the RbV_3Sb_5 and CsV_3Sb_5 monolayer. The TB parameters used for the RbV_3Sb_5 and CsV_3Sb_5 monolayer are $(\epsilon, t, t_2, \delta\epsilon, \delta t, \delta\epsilon_2) = (0.01, 0.42, 0.025, -0.0485, 0.0023, -0.0044)$ and $(0.036, 0.42, 0.005, -0.026, 0.0185, -0.012)$, respectively. Here, we introduce the additional onsite term $\frac{\delta\epsilon_2}{6}\left[(2\lambda_0 + 3\lambda_7 + \sqrt{3}\lambda_8) \otimes \sigma_0\right]$ to reproduce the exact energy of type-I VHSs in DFT results, which does not alter the symmetry. We also find that the type-II VHSs appear in the RbV_3Sb_5 and CsV_3Sb_5 monolayer, as shown in the DOS and TB energy contours in Fig. S2.

2.2. Phonon softening of the pristine phase

We perform a systematic study of the DFT phonon spectra of the monolayer. We find that there exists phonon softening in the pristine monolayer, as in the case of the bulk [6]. The DFT phonon spectra of the pristine monolayer for all three alkali atoms (Fig. S3) consistently show the phonon softening at the Γ and M points, associated with the distortion of V atoms. Here, the Γ (M) point corresponds to the one (other two) of the 2×2 $3Q$ CDW vectors in the folded BZ. These phonon spectra of the pristine monolayer clearly indicate structural instability, which justifies the consideration of the CDW in the monolayer.

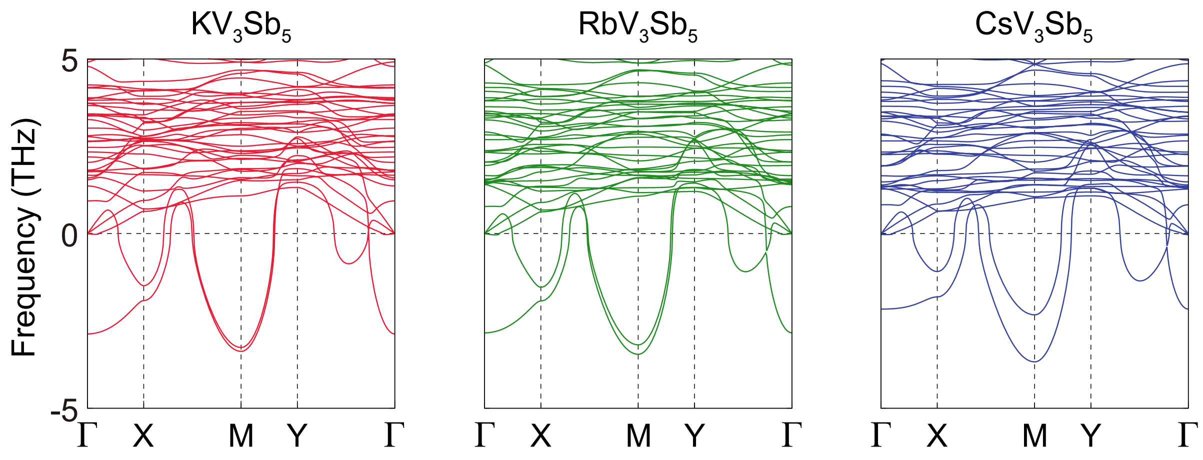

FIG. S3: **Phonon bands of the pristine phases for the AV_3Sb_5 monolayer family.** Here, the smearing factor $\sigma = 0.01$ eV is used.

2.3. Phonon spectra of the CDW phase

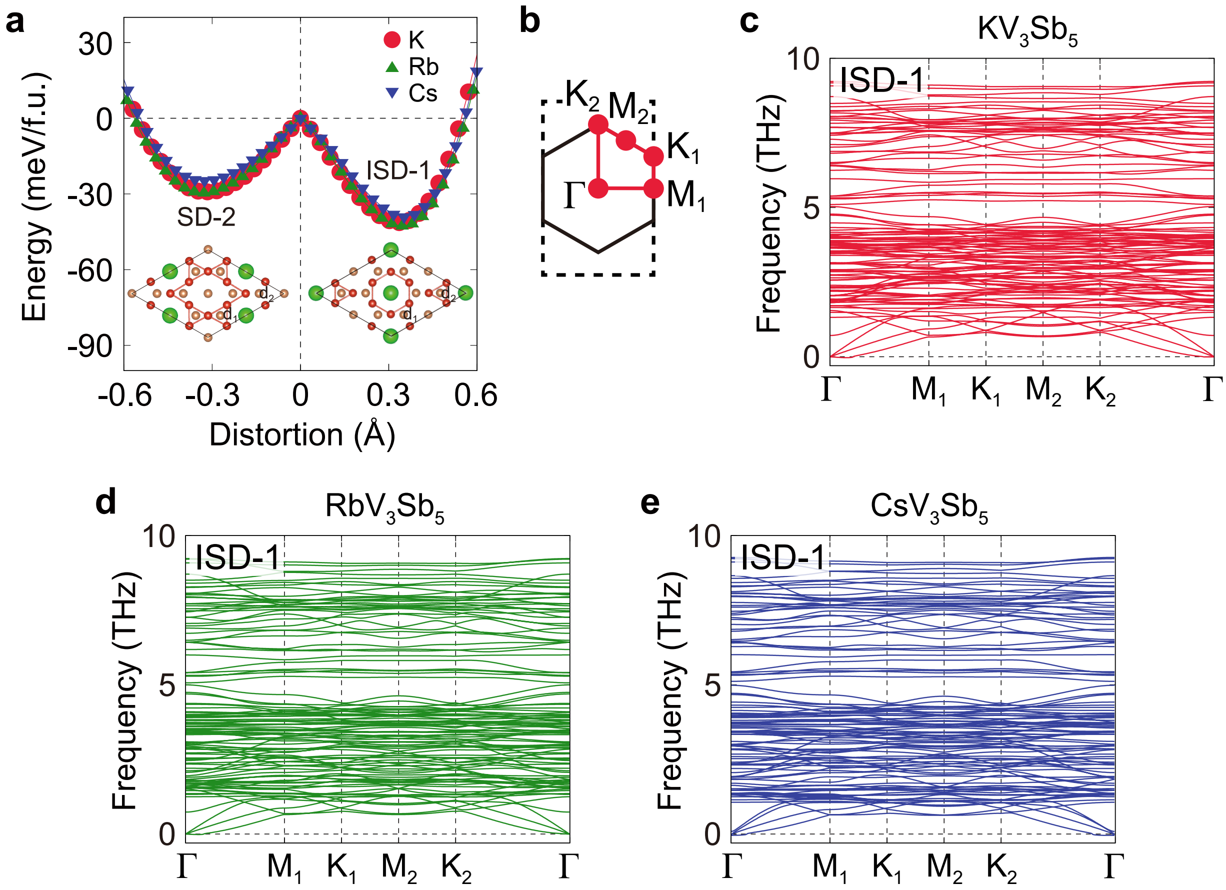

FIG. S4: **Energy profile and phonon band structures of the DFT ground state for the AV_3Sb_5 monolayer.** **a** Energy profile as a function of the distortion for the AV_3Sb_5 monolayer. The distortion is defined as $d \equiv d_1 - d_2$ where positive (negative) distortion corresponds to the 2×2 ISD-1 (SD-2) CDW phase whose geometry is displayed in the inset. **b** BZ and high symmetry points for the 2×2 unit cell. BZ for the $\sqrt{3} \times 1$ unit cell is drawn in a dashed line. **c-e** Phonon band structures for the ISD-1 phase of the AV_3Sb_5 monolayer. The 4×4 supercell with 9×9 k-points is used for the phonon band calculations.

This section provides the details of the DFT ground state and its stability in the AV_3Sb_5 monolayer. By extensively inspecting diverse CDW distortions, we find a stable structure in two dimensions. The 2×2 ISD-1 CDW phase is the DFT ground state and is dynamically stable for all three alkali atoms A = K, Rb, and Cs (Fig. S4). The 2×2 ISD-1 phase is energetically favored over the pristine counterpart by 41, 43, and 39 meV per formula unit for A = K, Rb, and Cs, respectively (Fig. S4**a**). It is noteworthy that the energy gain under the ISD distortion in the monolayer is much higher than that of the bulk structure (2, 8, and 15 meV per formula unit for A = K, Rb, and Cs, respectively), which implies the

enhanced instability of the 2D system. Moreover, nearly identical energy gains and CDW distortion magnitudes, irrespective of the type of alkali atoms A, for the ISD-1 phase reflect the strong 2D nature of V atoms in the AV_3Sb_5 monolayer. On the other hand, the 2×2 SD-2 CDW phase is metastable in the configuration space, as shown in the energy profile (Fig. S4**a**). We also find that the SD-1 and ISD-2 phases are unstable against their pristine counterparts, which indicates that the energetically preferred positions of alkali atoms are different between the ISD and SD phases.

Supplementary Note 3. Analysis on the VHS points

3.1. Sublattice character of VHS

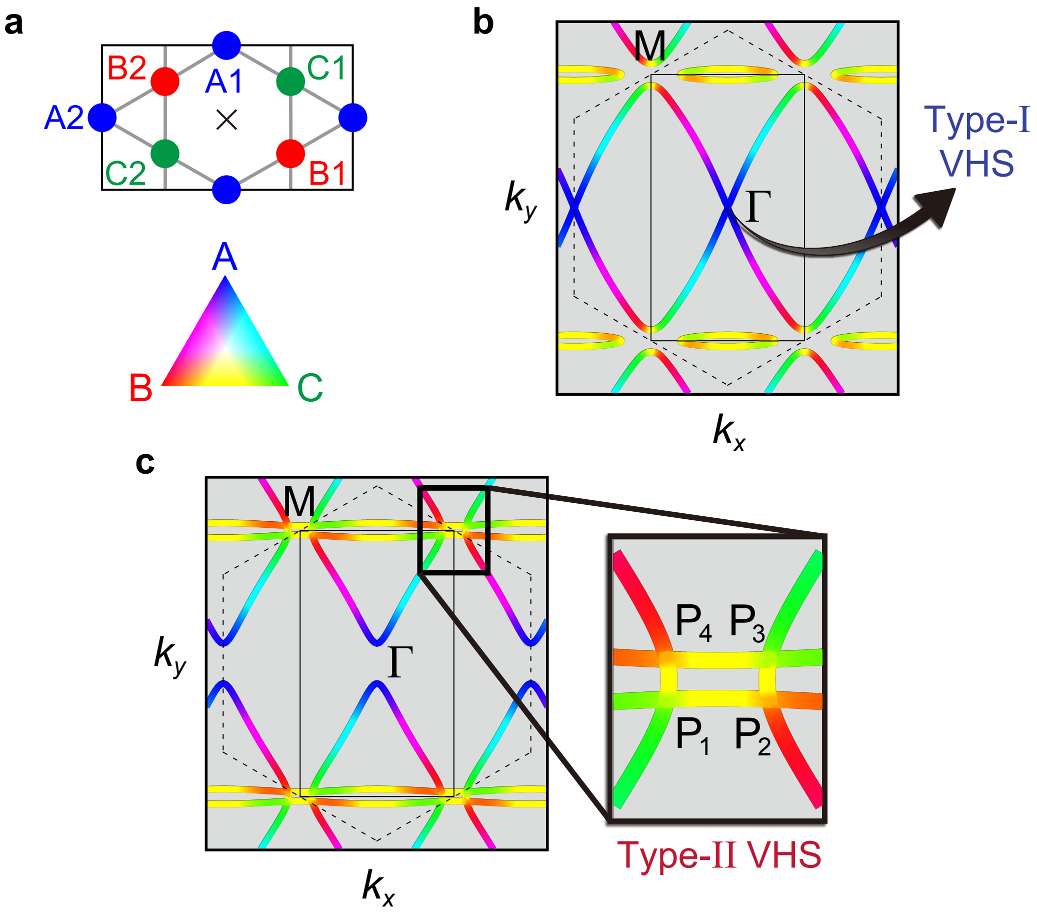

FIG. S5: **Sublattice character of the monolayer AV_3Sb_5. a** Six sublattices in the $\sqrt{3} \times 1$ unit cell for the TB model. The three different sublattices A, B, and C are colored in blue, red, and green, respectively, as shown in the color triangle. The position of alkali atoms is symbolically marked by $\times$ in the center of the unit cell. **b,c** Sublattice-projected energy contours of the TB bands for the KV_3Sb_5 monolayer. The contour curves in **b** and **c** correspond to $E = 9$ meV (type-I VHS) and -6 meV (type-II VHS), respectively. Thin black dashed (solid) lines show the BZ of the 1×1 ($\sqrt{3} \times 1$) unit cell.

Figure S5 shows the sublattice character of the VHS points in the monolayer. The sublattice character of the four type-II VHS points at P_i shows the mixing of B and C sublattices due to the symmetry lowering. Specifically, the ratio between two sublattices is B : C = 23% : 77% for P_1 and P_3 points and B : C = 77% : 23% for P_2 and P_4 points. Such sublattice-mixed type-II VHS points are in contrast to the sublattice-pure type-I VHS point

at Γ which consists of the single A sublattice.

3.2. Rearrangement of VHS

The appearance of type-I and type-II VHS points in the monolayer AV_3Sb_5 can be understood with the analogy of hybridization. Starting from the bulk system with $(D_{6h}, T_{1\times1})$ symmetries, the monolayer system is achieved by breaking both point group and translation symmetries, where the two states $\boldsymbol{k}$ and $\boldsymbol{k}+\boldsymbol{M}_1$ are mixed as a results of zone folding. Then, the type-II VHS is associated with the hybridization between two different M points at the Brillouin zone boundary (say, $\boldsymbol{k}=\boldsymbol{M}_2$ or $\boldsymbol{M}_3$) while the type-I VHS at Γ is just a shift of one M point (say, $\boldsymbol{k}=\boldsymbol{M}_1$), as illustrated in Fig.S6. The symmetry-lowering of the monolayer system hybridizes the two bands with different sublattice constitutions at the zone boundary, represented by different colors (red and green in Fig.S6) and produces the four type-II VHSs.

One of our observations is that the number of the resultant VHS points (four in our tight-binding model, Fig.S6) is not universal but crucially depends on the microscopic details of the system such as a Fermi-surface nesting condition parameter. To show these non-universal behaviors, it is useful to apply the effective two-band model, focusing on the bands which include the saddle points. One can perform the Taylor expansion near the saddle point $\boldsymbol{k}=\boldsymbol{M}_2+\boldsymbol{p}$, and obtain the effective 2×2 Hamiltonian,

$$\mathcal{H}^0_{\rm two}(\boldsymbol{p}) = \begin{pmatrix} \epsilon_2(\boldsymbol{p}) & 0 \\ 0 & \epsilon_3(\boldsymbol{p}) \end{pmatrix}, \quad \delta\mathcal{H}_{\rm two}(\boldsymbol{p}) = \begin{pmatrix} 0 & \Delta \\ \Delta & 0 \end{pmatrix}, \tag{2}$$

where $\epsilon_a(\boldsymbol{p})$ is expanded energy dispersion near M_a and Δ is an exemplified symmetry breaking term from $(T_{1\times1}, D_{6h})$ to $(T_{\sqrt{3}\times1}, D_{2h})$. We rotate the axes to diagonalize the Hessian matrix of the energy near the VHS of $(T_{1\times1}, D_{6h})$ system which gives

$$\epsilon_2(\boldsymbol{p}) = \frac{\alpha}{2} \begin{pmatrix} p_x & p_y \end{pmatrix} \begin{pmatrix} 1 & 0 \\ 0 & -\tan(\frac{\pi}{3}+\beta)^2 \end{pmatrix} \begin{pmatrix} p_x \\ p_y \end{pmatrix} + \cdots, \tag{3}$$

where the higher order terms, $O(\boldsymbol{p}^3)$, are omitted in $\cdots$. Here, the two coefficients $(\alpha>0, -\frac{\pi}{6}<\beta<\frac{\pi}{6})$ determine the behavior of VHS and Fermi-surface contours. For

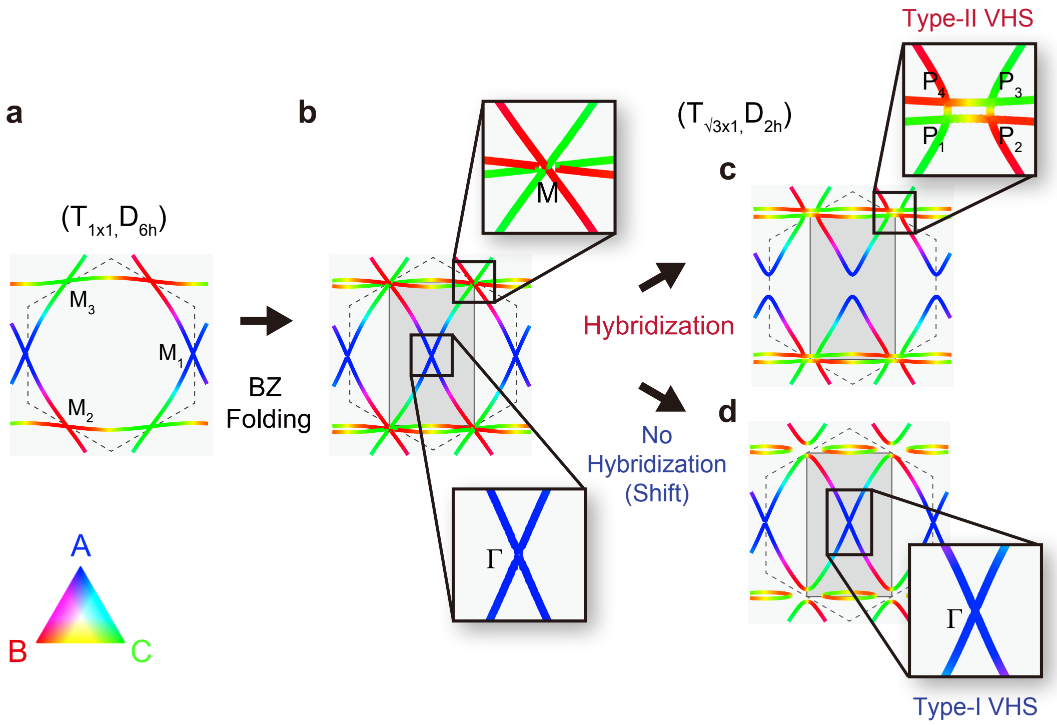

FIG. S6: **Schematic illustration of the type-I and -II VHS points in momentum space rearranged by symmetry lowering. a,b**. The VHS point distribution in $(T_{1\times1}, D_{6h})$ symmetry illustrated at $\sqrt{3}\times 1$ (1×1) unit cell which is indicated by solid (dashed) line, respectively. **c,d**. The hybridization due to symmetry-lowering into $(T_{\sqrt{3}\times1}, D_{2h})$ importantly affects to the type of VHS near M and Γ. More specifically, the two VHSs at M point become mixed and split into four type-II VHS points consisting of a mixed sublattice character, while the VHS at Γ still survives as a type-I VHS with a single sublattice character.

example, the parameter β encodes a Fermi-surface nesting condition, and its absolute value $|\beta|$ quantifies how far away from the perfect nesting condition ($\beta = 0$), which depends on the next nearest hopping parameter t_2. The original Hamiltonian $\mathcal{H}^0_{\rm two}$ enjoys a D_{6h} symmetry, and $\epsilon_3(\boldsymbol{p})$ is constructed by $\frac{2\pi}{3}$ rotation of $\epsilon_2(\boldsymbol{p})$,

$$\epsilon_2(\boldsymbol{p}) = \frac{\alpha}{2}\begin{pmatrix} p'_x & p'_y \end{pmatrix}\begin{pmatrix} 1 & 0 \\ 0 & -\tan(\frac{\pi}{3}+\beta)^2 \end{pmatrix}\begin{pmatrix} p'_x \\ p'_y \end{pmatrix} + \cdots, \tag{4}$$

with an unitary transformation,

$$\begin{pmatrix} p'_x \\ p'_y \end{pmatrix} = \begin{pmatrix} \cos(\frac{2\pi}{3}) & -\sin(\frac{2\pi}{3}) \\ \sin(\frac{2\pi}{3}) & \cos(\frac{2\pi}{3}) \end{pmatrix} = \begin{pmatrix} p_x \\ p_y \end{pmatrix}. \tag{5}$$

By diagonalizing the total effective two band Hamiltonian, $\mathcal{H}^0_{\rm two}(\boldsymbol{p}) + \delta\mathcal{H}_{\rm two}(\boldsymbol{p})$, we find

that the number of shifted VHS points becomes either 4 or 2, depending on the sign of the parameter $\beta > 0$ or $\beta < 0$, as illustrated in Fig. S7.

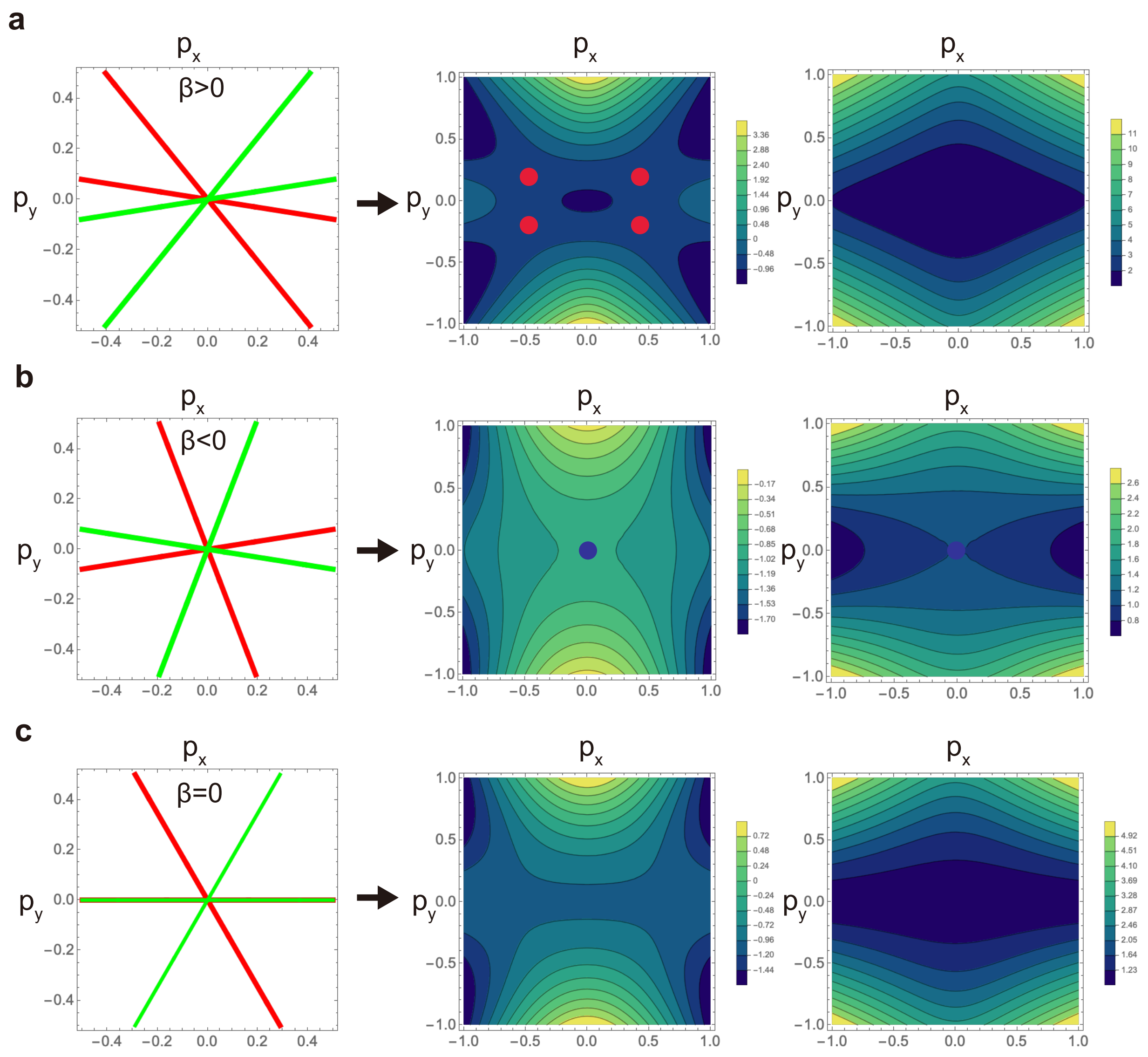

FIG. S7: **Number of VHSs depending on Fermi-surface nesting parameter β.** Based on the effective two-band model, Eq.4, we find that the number of VHSs is not universal but crucially depends on the microscopic details of VHSs. Figures **a-c** are for the different Fermi-surface nesting parameters, $(\beta = \frac{\pi}{20}, -\frac{\pi}{20}, 0)$. In leftmost panels of **a-c**, the the contours of the Fermi-surface before the hybridization $(\Delta = 0)$ are illustrated in the momentum space, (p_x, p_y), where $\boldsymbol{p} = \boldsymbol{k} - M$ is the momentum deviation from the original VHS points (M). The other two panels of **a-c** show the rearranged VHSs and Fermi contour of the energy dispersion of two bands after the hybridization $(\Delta = 0.1)$, where the plot legend is drawn with a unit of $\alpha/2$. For $\beta > 0$, the four type-II VHS points (red circles) newly appear at the shifted point, while the two type-I VHSs (blue circle) survive at M in each band for $\beta < 0$. In the fine-tuned case, $\beta = 0$, both derivative and Hessian matrix of energy are zero at $k_y = 0$ line, disappearing all VHS points and the higher order expansion may be essential to examine the possibility of the high-order VHSs.

Supplementary Note 4. Sublattice resolved susceptibility

We demonstrate the sublattice interference in the monolayer system by calculating the sublattice resolved susceptibility, following the previous literature [7]. The explicit form of the susceptibility is given by

$$\chi^{l_1,l_2}(\boldsymbol{q}) \equiv -\sum_{\boldsymbol{k},\mu,\nu} a_\nu^{l_1}(\boldsymbol{k}+\boldsymbol{q}) a_\mu^{l_1}(\boldsymbol{k})^* a_\nu^{l_2}(\boldsymbol{k}+\boldsymbol{q})^* a_\mu^{l_2}(\boldsymbol{k}) \left[\frac{n_F(E_\mu(\boldsymbol{k})) - n_F(E_\nu(\boldsymbol{k}+\boldsymbol{q}))}{E_\mu(\boldsymbol{k}) - E_\nu(\boldsymbol{k}+\boldsymbol{q})}\right] \quad (6)$$

with sublattice index, (l_1, l_2), and band index, (μ, ν). Here, the Fermi distribution, $n_F(\epsilon)$, and the l_i-th component of the eigenvector of the μ band, $a_\mu^{l_i}(\boldsymbol{k})$, are introduced. The eigenvector of the largest eigenvalue of $\chi^{l_1,l_2}(\boldsymbol{q})$ around M point encode the sublattice interference effects. For example, in the two opposite limits, the sublattice weights of (A,B,C) sublattice are given by either (0,0.5,0.5), (0.5,0,0.5) or (0.5,0.5,0) in the case displaying a perfect sublattice interference, while the values become either (1,0,0), (0,1,0) or (0,0,1) in the perfectly sublattice interference absent limit, as illustrated in Fig. S8**a**.

Based on the tight-binding model of monolayer AV_3Sb_5 introduced in the main text, we found that the sublattice interference is suppressed, but it is not absent. The largest eigenvalues of the susceptibility at the M point have the sublattice weights, (0.482,0.258,0.258) and (0.476,0.262,0.262), for the type-I, type-II VHS filling, which are significantly modified from the perfectly sublattice interference absent case (Fig. S8**b,c**). Hence, we can argue that V still plays a crucial role in offering new physics in monolayer AV_3Sb_5, as in the bulk system.

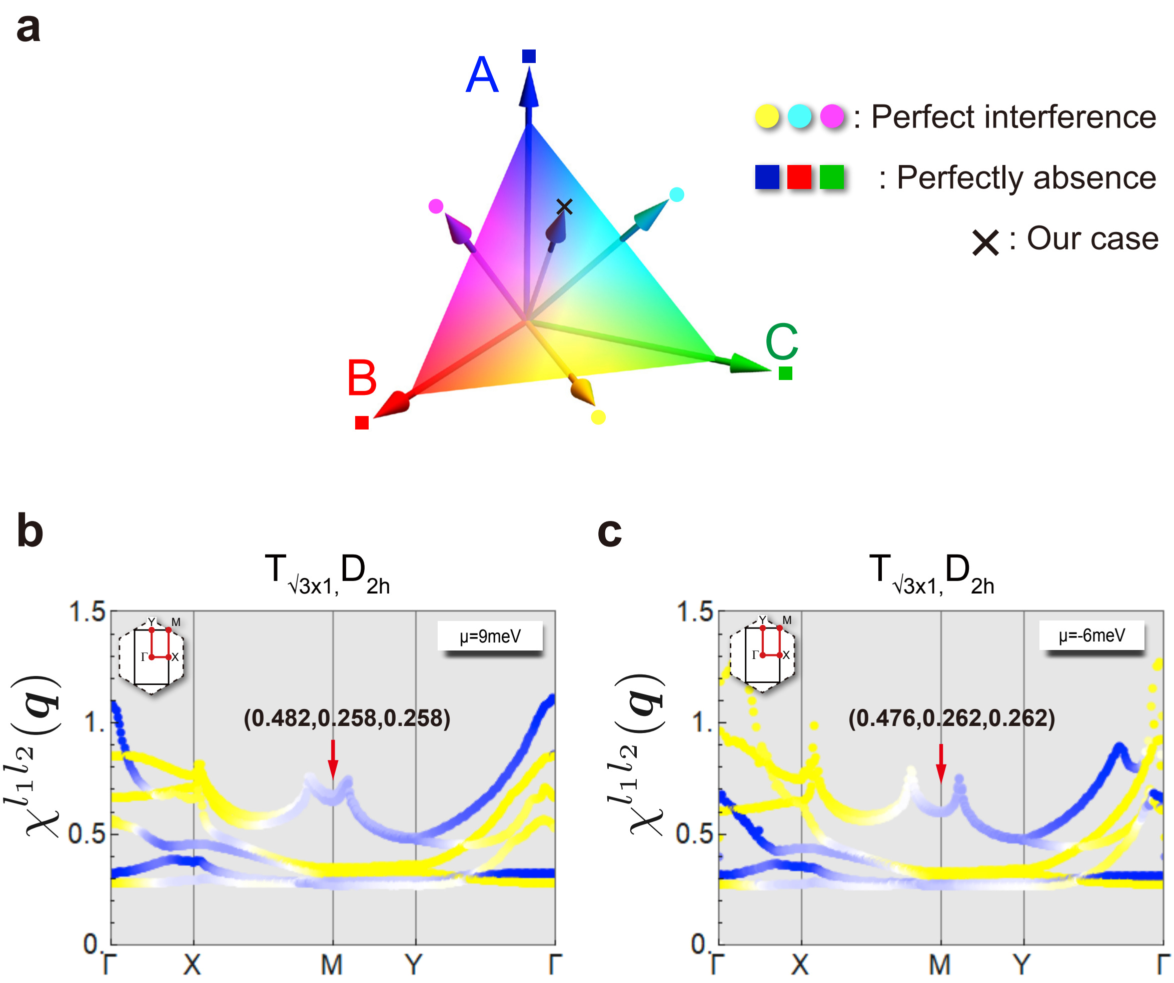

FIG. S8: **Sublattice-resolved bare susceptibility of AV_3Sb_5 systems.** **a.** The three different sublattices A, B, and C are colored in blue, red, and green, respectively. It is useful to utilize the eigenvector associated with the largest eigenvalues of $\chi^{l_1,l_2}(\boldsymbol{q})$ to quantify the sublattice interference. For example, the sublattice weight would be either (0,0.5,0.5), (0.5,0,0.5) or (0.5,0.5,0) in the limit displaying a perfect sublattice interference, while the values become either (1,0,0), (0,1,0) or (0,0,1) in the perfectly sublattice interference absent limit. The sublattice character of the monolayer AV_3Sb_5 system corresponds to the intermediate case between two limits. **b-c.** The eigenvalues of bare susceptibility matrix $\chi^{l_1,l_2}(\boldsymbol{q})$ and their sublattice characters are carried out based on our tight-binding Hamiltonian at $\beta = 1000$. (b) is for the AV_3Sb_5 systems at type-I VHS filling, while (c) is at type-II VHS filling. Focusing on the sublattice character of largest eigenvalues of $\chi^{l_1,l_2}(\boldsymbol{q} = M)$, we find that (0.482,0.258,0.258), and (0.476,0.262,0.262) for b,c, respectively.

Supplementary Note 5. Detailed phonon analysis

5.1. Leading CDW instability of the pristine phase

In this subsection, we show that the 2×2 CDW instability is a leading instability by performing energy profile analysis along the various negative phonon modes. Fig. S9**a** shows the phonon band structure of the pristine monolayer. The negative modes appear at a broad region in the momentum space. A close inspection reveals that they appear delocalized as they are localized at multiple $\boldsymbol{k}$-point, including high symmetry points Γ, M, and X. Among these, the negative energies at the Γ point arise from the folding of the M point of the 1×1 unit cell to the $\sqrt{3} \times 1$ unit cell, responsible for the 2×2 instability. We evaluated the total energy profiles for the reconstructions driven by the negative phonon modes at high symmetry X and M points as well as the nesting vectors q_1 and q_2 mediating the type-II VHS points (Fig. S9**b**), in which the latter is to consider the incommensurate CDWs. Here, we choose q_1 and q_2 to be commensurate, namely $(\frac{1}{6}, 0, 0)$ and $(0, \frac{1}{10}, 0)$, respectively, which are the closest rational numbers to the incommensurate nesting vectors mediating the type-II VHS points (see also Fig. 3**a** in the main text). The figure clearly shows that the reconstructions driven by X, q_1, and q_2 are less stable than that of M. Thus, the 2×2 reconstruction is energetically favored over the other reconstructions captured in the negative phonon energies of the monolayer.

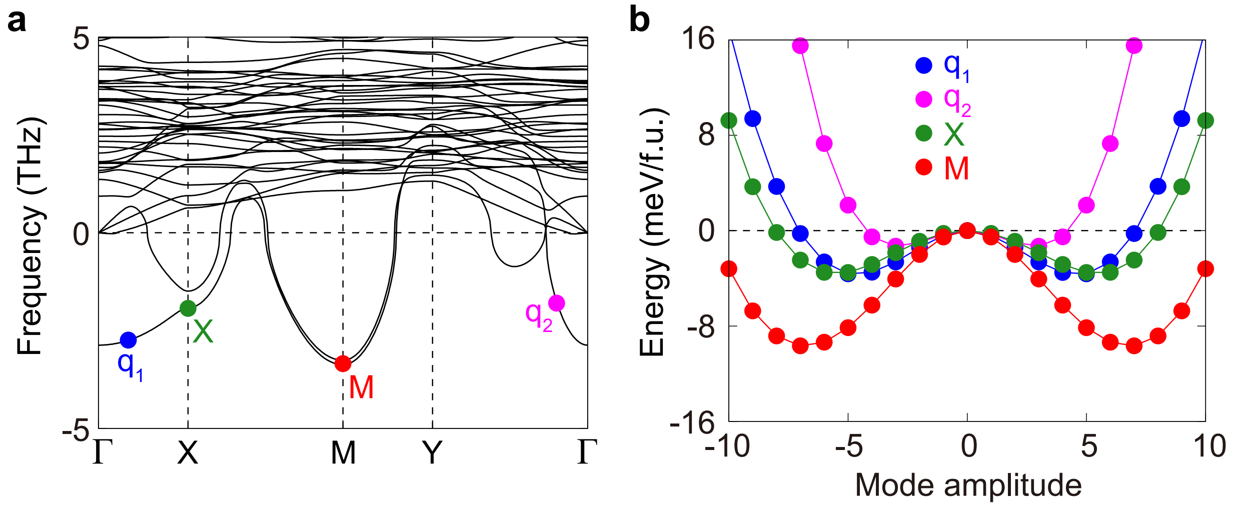

FIG. S9: **Negative phonon mode analysis of the $A\mathrm{V_3Sb_5}$ monolayer.** **a** Phonon bands of the pristine $\mathrm{KV_3Sb_5}$ monolayer at the smearing factor $\sigma = 0.1$ eV. Here, $q_1 = \frac{1}{6}\mathbf{b}'_1$ and $q_2 = \frac{1}{10}\mathbf{b}'_2$ ($\mathbf{b}'_1$ and $\mathbf{b}'_2$ are the reciprocal lattice vectors of the $\sqrt{3} \times 1$ unit cell) are the closest commensurate vectors to the incommensurate nesting vectors mediating the type-II VHS points (see also Fig. 3a in the main text). **b** Energy profiles (per formula unit) as a function of the amplitude of the phonon mode for various negative energy states. The corresponding energy and momenta are indicated in **a**. We stress that the minimum of total energy appears at M (~ -9.7 meV/f.u), which corresponds to the 2×2 CDW reconstruction.

5.2. Correction of artificial negative phonon modes of the ISD-1 phase

In this subsection, we demonstrate the correction of the artificial negative phonon modes of the ISD-1 phase. We calculate the phonon bands by increasing the supercell size from 4×4 to 8×8 (Fig. S10), which allows us to get more accurate results without the negative branches. Thus, we conclude that the phonon bands show the stability of the 2×2 reconstruction in the D_{2h} monolayer.

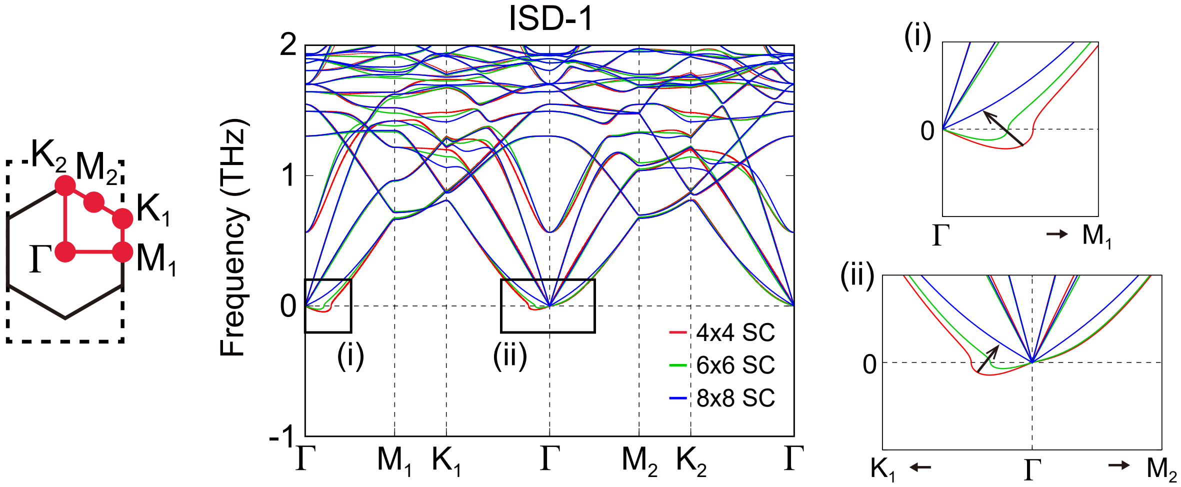

FIG. S10: **Convergence test of the negative phonon energies of the ISD-1 structure.** The phonon bands of the ISD-1 structure are calculated using three different supercells (SCs): 4×4, 6×6, and 8×8. The erroneous negative energies, which appears in the 4×4 and 6×6 SC calculations, converge to positive ones as we increase the computational accuracy using a large SC. The insets (i) and (ii) display the magnified views of the boxed regions near Γ. The arrows indicate the converging behavior of the negative branches (from 4×4 to 8×8 SC). The left panel depicts the Brillouin zone (solid line) and the corresponding high-symmetry points of the 2×2 unit cell.

Supplementary Note 6. Details on various CDW orders

6.1. Anisotropic 2×2 $3Q$ CDW order

The lowered symmetries of the monolayer can favor the $3Q$ SD and ISD phases as they respect the lower symmetries. A close look at the SD and ISD distortion of the monolayer reveals that they adapt themselves into the lowered symmetries by losing C_3 symmetries and being split into the doublets, which we refer to as SD-1 and SD-2 and ISD-1 and ISD-2, respectively. As delineated in Fig. S11, the adjacent two vanadium atoms have different bonding lengths between the AB and BC sublattices. These patterns are unlike the D_{6h} bulk counterpart, in which the bonding lengths are the same between the adjacent V atoms. Since these types of distortion respect the $\sqrt{3} \times 1$ symmetries, it is likely that the monolayer symmetry favors them.

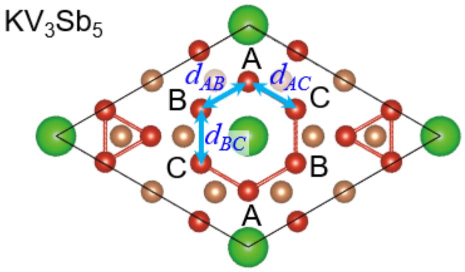

Bond Length ($\AA$)	Monolayer	Bulk
d_{AB}	2.698	2.675
d_{AC}	2.698	2.675
d_{BC}	2.646	2.675

FIG. S11: **Bond lengths of the ISD-1 phase in the monolayer.** Sublattice indices A, B, and C (for hexagons) are depicted on left. The calculated bond lengths between the sublattices are given in Table (in Å). In contrast to the isotropic CDW order of the bulk counterpart, the CDW in the monolayer has anisotropic bond lengths, such that $d_{BC} < d_{AB} = d_{AC}$.

6.2. Band structures of ISD-1 and SD-2 CDWs

We calculate the band structure of the 2×2 ISD-1 and SD-2 CDWs of the AV_3Sb_5 monolayer for $A =$ K, Rb, Cs, as shown in Fig. S12. Due to the symmetry breaking, M_1 and M_2 (or K_1 and K_2) have no longer the same energy.

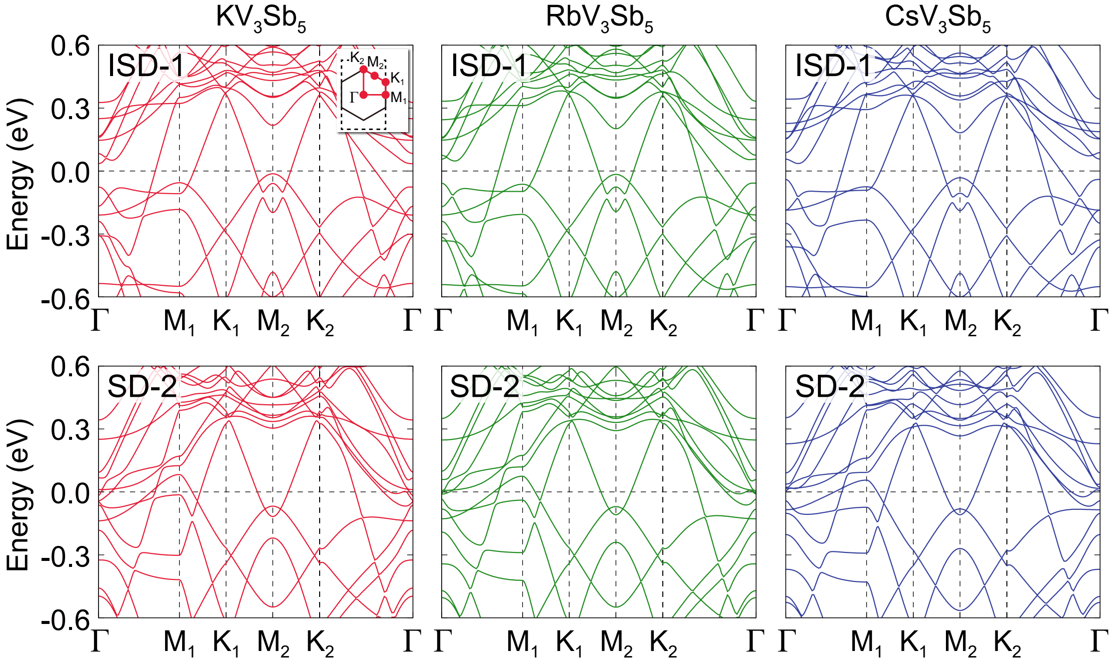

FIG. S12: **The band structures of symmetry-breaking phases in the monolayer AV$_3$Sb$_5$.** Left, middle, and right panels indicate the band structures of ISD-1 and SD-2 phases for K, Rb, and Cs systems, respectively.

6.3. TRSB CDW: Comparison with the D_{6h} bulk

In this section, we compare the TRSB-1 and TRSB-2 CDWs of the D_{2h} monolayer with their D_{6h} bulk counterpart (Fig. S13). The complex CDW introduced in arXiv:2110.06266 (Ref. [8]) can be considered as a bulk counterpart of the TRSB-1 and TRSB-2 CDWs that we studied in the monolayer. As compared in Fig. S13, the current patterns of our TRSB-1 and TRSB-2 are the same as those of the complex CDW order of Ref. [8] with the magnetic fluxes threading in all the hexagons and triangles. Notably, the Chern Fermi pockets are featured in our system as well, realizing the phase referred to as a doped orbital Chern insulator by Zhou and Wang.

We explicitly show that the Chern numbers are distributed differently over the bands near the Fermi level between the D_{2h} monolayer and the D_{6h} bulk, as delineated in Fig. S13. In particular, the different distributions of the Berry curvatures on the Fermi surface are found near the $M1$ and $M2$ (as well as $K1$ and $K2$) points, which can be mainly attributed to distinct parameters that are chosen such that they depict the absence of the C_{3z} rotational symmetry of the monolayer. In detail, the threading flux values of the TRSB phase in Ref. [8] are different from our TRSB-1 and TRSB-2 phases (Table. S2). The complex CDW

phase in Ref. [8] has flux values as $\phi_{1,\ldots,4} = (0.840, -0.113, -0.291, 0.014)\pi$ while our TRSB-1 and TRSB-2 phases have the flux values as $\phi_{1,\ldots,4} = (0.318, -0.103, -0.159, 0.051)\pi$ and $\phi_{1,\ldots,4} = (0.313, -0.108, -0.157, 0.054)\pi$, respectively.

Correspondingly, distinct anomalous Hall conductivities were calculated between the bulk and monolayer systems. The bulk system has the anomalous Hall conductivity of around $-1.1\ \frac{e^2}{h}$, whereas the monolayer in TRSB-1 and TRSB-2 phases lead to -0.26 and -0.06 $\frac{e^2}{h}$, respectively, Here, e and h are electronic charge and the Plank constant, respectively. This difference in the anomalous Hall conductivities should be of crucial importance for distinguishing the bulk and monolayer systems.

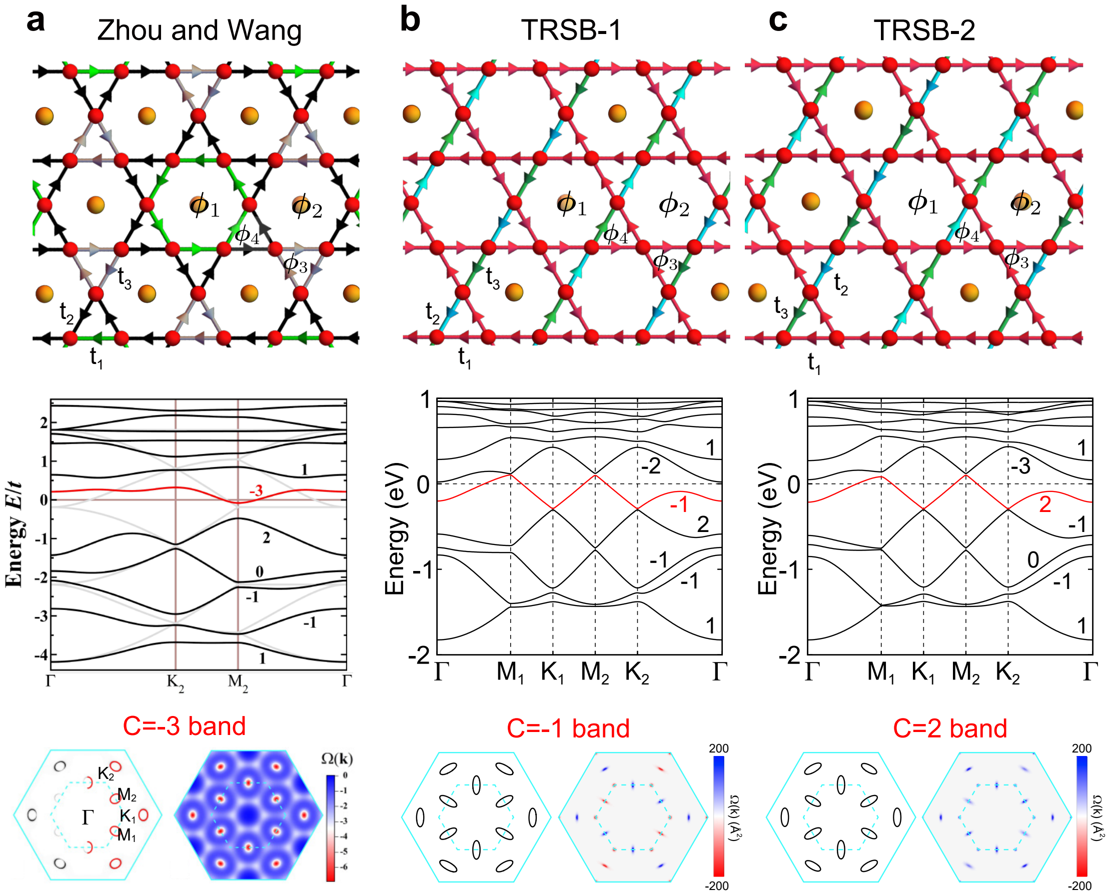

FIG. S13: **Current pattern, band structure, Fermi surface, and Berry curvature of various TRSB phases. a** Current pattern, band structure, Fermi surface, and Berry curvature of the TRSB phase studied in Zhou and Wang [8]. **b,c** Current pattern, band structure, Fermi surface, and Berry curvature of the TRSB-1 and TRSB-2 phases were studied in this work. The magnitudes of threading fluxes ϕ_1, ϕ_2, ϕ_3, and ϕ_4 as well as the hopping parameters are given in Table S2. The different hopping parameters are displayed in different colors. Chern numbers of each band are displayed in the band structures. Energy contours and Berry curvature distribution of the band lying at the Fermi level (colored in red in the band structures) are drawn in the bottom panel.

TABLE S2: **Magnetic fluxes and complex hopping parameters of the TRSB phases in Fig. S13.** The unit of hopping parameters is the electron volt (eV). The ± sign of the imaginary hopping constants denotes the direction of the bond current depicted in Fig S13.

Model	ϕ_1	ϕ_2	ϕ_3	ϕ_4	t_1	t_2	t_3
Ref. [8]	0.840π	-0.113π	-0.291π	0.014π	$0.345 \pm 0.162i$	$0.499 \pm 0.123i$	$0.349 \pm 0.110i$
TRSB-1	0.318π	-0.103π	-0.159π	0.051π	$0.42 \pm 0.07i$	$0.41 \pm 0.07i$	$0.43 \pm 0.07i$
TRSB-2	0.313π	-0.108π	$\pm 0.157\pi$	0.054π	$0.42 \pm 0.07i$	$0.41 \pm 0.07i$	$0.43 \pm 0.07i$

Supplementary Note 7. Details on tight-binding Hamiltonian

7.1. Notation

In this section, we provide detailed information on notations about our fermionic systems. We introduce the three-dimensional Gamma matrices (λ_a),

$$\lambda_1 = \begin{pmatrix} 0 & 1 & 0 \\ 1 & 0 & 0 \\ 0 & 0 & 0 \end{pmatrix}, \ \lambda_2 = \begin{pmatrix} 0 & 0 & 1 \\ 0 & 0 & 0 \\ 1 & 0 & 0 \end{pmatrix}, \ \lambda_3 = \begin{pmatrix} 0 & 0 & 0 \\ 0 & 0 & 1 \\ 0 & 1 & 0 \end{pmatrix}, \ \lambda_4 = \begin{pmatrix} 0 & -i & 0 \\ i & 0 & 0 \\ 0 & 0 & 0 \end{pmatrix},$$

$$\lambda_5 = \begin{pmatrix} 0 & 0 & -i \\ 0 & 0 & 0 \\ i & 0 & 0 \end{pmatrix}, \ \lambda_6 = \begin{pmatrix} 0 & 0 & 0 \\ 0 & 0 & -i \\ 0 & i & 0 \end{pmatrix}, \lambda_7 = \begin{pmatrix} 1 & 0 & 0 \\ 0 & -1 & 0 \\ 0 & 0 & 0 \end{pmatrix}, \ \lambda_8 = \frac{1}{\sqrt{3}}\begin{pmatrix} 1 & 0 & 0 \\ 0 & 1 & 0 \\ 0 & 0 & -2 \end{pmatrix},$$

with $\lambda_0 = I_3$. It is convenient to introduce the four dimension matrices (M_a) to express the sublattice subspace in 2×2 unit cell,

$$M_1^{\pm} = \frac{1}{2}\left[(I_2 \pm \sigma_x) \otimes I_2\right], \ M_2^{\pm} = \frac{1}{2}\left[I_2 \otimes (I_2 \pm \sigma_x)\right], \ M_3^{\pm} = \frac{1}{2}\left[\pm I_2 \otimes I_2 + \sigma_x \otimes \sigma_x\right],$$

with $M_0 = I_4$ and define twelve dimensional matrices ($L_a^0, L_a^{\pm}$),

$$L_a^0 = \lambda_a \otimes M_0, \quad L_a^{\pm} = \begin{cases} \lambda_a \otimes M_1^{\pm} & \text{for } a = 1, 4, \\ \lambda_a \otimes M_2^{\pm} & \text{for } a = 2, 5, \\ \lambda_a \otimes M_3^{\pm} & \text{for } a = 3, 6. \end{cases} \tag{7}$$

7.2. Tight-binding Hamiltonian in 2×2 unit cell

This section provides the details on tight-binding (TB) Hamiltonian in 2×2 unit cell. Introducing the twelve-component spinor, the Hamiltonian becomes $H_0 = \sum_{\boldsymbol{k},\sigma} \tilde{\Psi}^{\dagger}_{\boldsymbol{k}\sigma}\mathcal{H}_0(\boldsymbol{k})\tilde{\Psi}_{\boldsymbol{k}\sigma}$ with $\tilde{\Psi}^T_{\boldsymbol{k}} = (\tilde{A}^T_{\boldsymbol{k}}, \tilde{B}^T_{\boldsymbol{k}}, \tilde{C}^T_{\boldsymbol{k}})$ and $\tilde{\alpha}^T_{\boldsymbol{k}} = (\alpha_{1,\boldsymbol{k}}, \alpha_{2,\boldsymbol{k}}, \alpha_{3,\boldsymbol{k}}, \alpha_{4,\boldsymbol{k}})$. The site of the sublattice index of the 2×2 unit cell and the corresponding Brillouin zone are illustrated in Fig. S14. Our

minimal TB Hamiltonian may be decomposed as four different terms,

$$\mathcal{H}_0(\boldsymbol{k}) = \mathcal{H}_{NN}(\boldsymbol{k}) + \mathcal{H}_{NNN}(\boldsymbol{k}) + \mathcal{H}_1(\boldsymbol{k}) + \mathcal{H}_2(\boldsymbol{k}), \quad (8)$$

with

$$\begin{aligned}\mathcal{H}_{NN}(\boldsymbol{k}) &= -t\,[c_1\lambda_1 \otimes (I_2+\sigma_x)\otimes I_2 + s_1\lambda_4 \otimes (-I_2+\sigma_x)\otimes I_2 \\ &\quad + c_2\lambda_2 \otimes I_2 \otimes (I_2+\sigma_x) \otimes s_2\lambda_5 \otimes I_2 \otimes (-I_2+\sigma_x) \\ &\quad + c_3\lambda_3(I_2\otimes I_2 + \sigma_x\otimes\sigma_x) + s_3\lambda_6(I_2\otimes I_2 - \sigma_x\otimes\sigma_x)\,] + \epsilon I_{12},\end{aligned}$$

$$\begin{aligned}\mathcal{H}_{NNN}(\boldsymbol{k}) &= -t_2\,[c_4\lambda_1 \otimes (I_2+\sigma_x)\otimes \sigma_x + s_4\lambda_4 \otimes (-I_2+\sigma_x)\otimes \sigma_x \\ &\quad + c_5\lambda_2 \otimes \sigma_x \otimes (I_2+\sigma_x) \otimes s_5\lambda_5 \otimes \sigma_x \otimes (I_2-\sigma_x) \\ &\quad + c_6\lambda_3(I_2\otimes \sigma_x + \sigma_x\otimes I_2) + s_6\lambda_6(I_2\otimes \sigma_x - \sigma_x\otimes I_2)\,],\end{aligned}$$

$$\mathcal{H}_1(\boldsymbol{k}) = \frac{\delta\epsilon}{6}\left[(2\lambda_0 + 3\lambda_7 + \sqrt{3}\lambda_8)\otimes\sigma_z\otimes\sigma_z\right],$$

$$\mathcal{H}_2(\boldsymbol{k}) = -\delta t\,[c_3\lambda_3(I_2\otimes I_2 + \sigma_x\otimes\sigma_x) + s_3\lambda_6(I_2\otimes I_2 - \sigma_x\otimes\sigma_x)]\,.$$

The notations $(c_i, s_i) = (\cos\boldsymbol{k}\cdot\boldsymbol{r}_i, \sin\boldsymbol{k}\cdot\boldsymbol{r}_i)$ with $\boldsymbol{r}_1 = \frac{1}{2}(\sqrt{3},1)$, $\boldsymbol{r}_2 = \frac{1}{2}(\sqrt{3},-1)$, $\boldsymbol{r}_3 = (0,1)$, $\boldsymbol{r}_4 = \boldsymbol{r}_2 - \boldsymbol{r}_3$, $\boldsymbol{r}_5 = \boldsymbol{r}_1 + \boldsymbol{r}_3$, and $\boldsymbol{r}_6 = \boldsymbol{r}_1 + \boldsymbol{r}_2$ is used. Here, t, t_2, ϵ are nearest-neighbor hopping, next nearest-neighbor hopping, onsite energy and δt, $\delta\epsilon$ are anisotropic hopping,

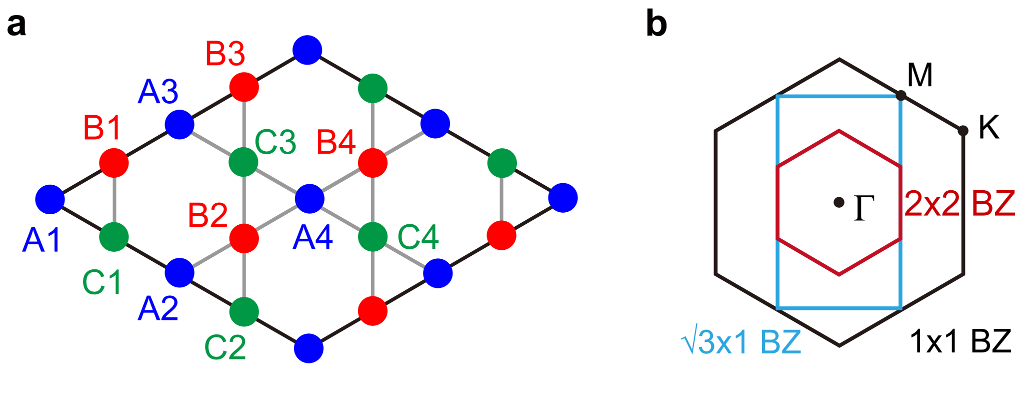

FIG. S14: 2×2 **unit cell and its Brillouin zone for TB model of the monolayer AV$_3$Sb$_5$.** **a** Twelve sublattices in the 2×2 unit cell for the TB model. T he three different sublattices A, B, and C are colored in blue, red, and green, respectively. **b** The BZs of the 2×2,$\sqrt{3}\times1$, and 1×1 unit cell are illustrated by red, cyan, and black lines, respectively.

anisotropic onsite energy which break symmetries from $\mathcal{T}_{1\times1}$ D_{6h} to $\mathcal{T}_{\sqrt{3}\times1}$ D_{2h}. The tight-binding parameter set $(\epsilon, t, t_2, \delta\epsilon, \delta t) = (0.01, 0.42, 0.03, -0.033, 0.01)$ is consistent with DFT band structures of KV_3Sb_5 and used in most of our discussion unless otherwise stated.

7.3. Justification of the tight-binding model

We augmented the justification of our minimal model in terms of the relevant orbitals contributing to bands around the Fermi level. The orbital-projected bands shown in Fig. S15 reveal that the VHS band described by our model mainly comprises three d-orbitals of V atoms: d_{xy}, $d_{3z^2-r^2}$, and $d_{x^2-y^2}$. Our group-theoretical analysis of the DFT Bloch state at M shows that the VHS state is in the A_g irreducible representation of the little group of M. This indicates that the three d orbitals of V are hybridized to form a A_g orbital, based on which our minimal model is constructed. Therefore, our minimal model was able to reproduce the DFT VHS band successfully.

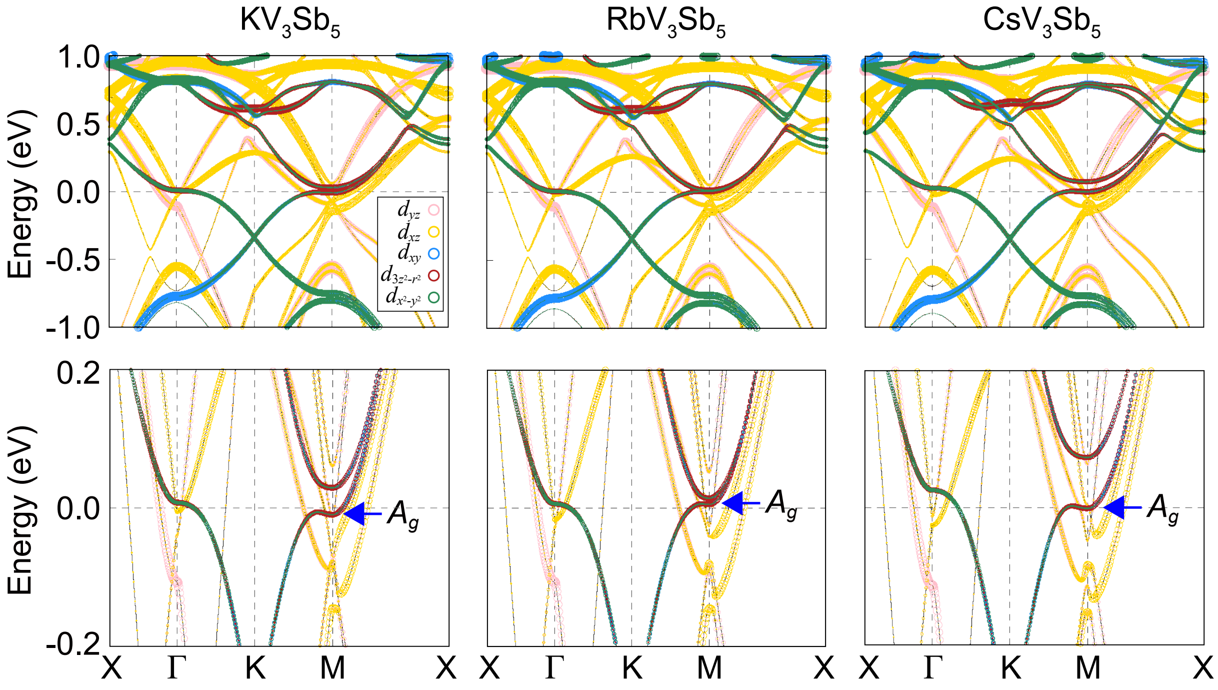

FIG. S15: **Orbital-projected band structures of the AV_3Sb_5 monolayer.** d_{yz}, d_{xz}, d_{xy}, $d_{3z^2-r^2}$ and $d_{x^2-y^2}$ orbitals of V atoms are projected on the band structure where the magnitude of open circles is proportional to the projected weight.

Our minimal model only considers the A_g VHS and thus other two VHSs are excluded because they are relatively away from the Fermi level. Thus, they should be less important

in describing the low-energy electronic property of the AV_3Sb_5 monolayer. This is, in fact, a unique feature of the monolayer. Unlike the bulk materials [9], the monolayer always hosts the A_g VHS nearest to the Fermi level irrespective of the type of alkali metal A (Fig. S15). Thus, our minimal model generically captures the low-energy electronic structure of the AV_3Sb_5 monolayer without the inclusion of the other VHS bands.

Supplementary Note 8. Details on mean-field analysis

8.1. Model and strategy

We carry out the zero temperature mean-field phase diagram following the steps.

(i) We consider the non-interacting Hamiltonian H_0 incorporating with density-density interactions $H_{\rm int}$ whose form may be expressed as

$$H_{\rm int} = U \sum_{\boldsymbol{R}} \sum_{\alpha_i} n_{\boldsymbol{R},\alpha_i \uparrow} n_{\boldsymbol{R},\alpha_i \downarrow} + V \sum_{\boldsymbol{R}} \sum_{\substack{\langle \alpha_i, \beta_j \rangle \\ \sigma,\sigma'}} n_{\boldsymbol{R},\alpha_i,\sigma} n_{\boldsymbol{R},\beta_j,\sigma'} \equiv U_{\rm int} + V_{\rm int}, \tag{9}$$

where ($\alpha \in \{A, B, C\}, i \in \{1, 2, 3, 4\}$) and $\sigma \in \{\uparrow, \downarrow\}$ are the indices for sublattice and spin, respectively.

(ii) We first check the CDW instability under the specific choice of CDW configurations (see Table S3 and Fig. 3**c**). We construct the CDW mean-field Hamiltonian in 2×2 unit cell which is convenient to see the physics after translational-symmetry breaking. Introducing a CDW order (Φ), the mean-field Hamiltonian is given by

$$\delta H_{\rm MF}[\Phi] = \Phi \sum_{\boldsymbol{k},\sigma} \tilde{\Psi}^\dagger_{\boldsymbol{k}\sigma} \begin{pmatrix} 0 & O_1(\boldsymbol{k}) & O_2(\boldsymbol{k}) \\ O_1(\boldsymbol{k})^\dagger & 0 & O_3(\boldsymbol{k}) \\ O_2(\boldsymbol{k})^\dagger & O_3(\boldsymbol{k})^\dagger & 0 \end{pmatrix} \tilde{\Psi}_{\boldsymbol{k}\sigma}, \tag{10}$$

with the twelve-component spinor, $\tilde{\Psi}^T_{\boldsymbol{k}}$. After evaluating the ground state of the mean-field Hamiltonian $|G; \Phi\rangle$ and its energy expectation value $E[\Phi; U, V]$, the phase diagram associated with CDWs can be obtained as a function of interaction strengths.

(iii) We further consider the SC instability. The superconducting pairing gap functions are classified based on the point group and fermionic anticommuting relation (see Table S4). Among them, we consider the spin-singlet pairings whose mean-field Hamiltonian is given by

$$\delta H_{\rm MF}[\Delta] = \Delta \left[\sum_{\boldsymbol{k}} \tilde{\Psi}^\dagger_{\boldsymbol{k}\uparrow} \hat{\Delta}(\boldsymbol{k}) \tilde{\Psi}^*_{\boldsymbol{k}\downarrow} + \text{C.C.} \right], \tag{11}$$

where the overall pairing amplitude (Δ) is chosen as a real number. Performing the same process, we obtain the full phase diagram including SC phases.

We obtain 161 data points of the ground state energy $E[\phi_i]$ where the order parameter range is $\phi_i \in [-0.08, 0.08]$. The integrations at given ϕ_i are evaluated in the momentum space with 80×80 k-points. In the following two sections, we provide detailed information on the ground states of the mean-field Hamiltonians of CDW ($\phi = \Phi$) and SC ($\phi = \Delta$).

8.2. Charge density wave (CDW)

This section provides details on the CDW order parameters and their mean-field theories. We start with the mean-field Hamiltonian density in 2×2 unit cell,

$$\delta\mathcal{H}_{\mathrm{MF}}[\Phi; \boldsymbol{k}] = \Phi \begin{pmatrix} 0 & O_1 & O_2 \\ O_1^\dagger & 0 & O_3 \\ O_2^\dagger & O_3^\dagger & 0 \end{pmatrix}, \tag{12}$$

where Φ is a CDW order parameter. The order parameter couples with the charge distribution of the V ions whose pattern is encoded in the bond matrix $O_a(\boldsymbol{k})$, where the index $a = 1, 2, 3$ labels AB, AC, BC bond. Strikingly, the number of CDW modes of the monolayer system ($\mathcal{T}_{\sqrt{3}\times 1}$ D_{2h}) is doubled compared to the bulk system ($\mathcal{T}_{1\times 1}$ D_{6h}). It is understood by the quotient group of two translational symmetries $\mathbb{Z}_2 = \mathcal{T}_{1\times 1}/\mathcal{T}_{\sqrt{3}\times 1}$ and this $\mathbb{Z}_2$ factor specifies the doublet CDW. Hereafter, the two states in the CDW doublet are denoted as "CDW-1/2", in which they are connected to each other by $\mathcal{T}_{\sqrt{3}\times 1}$ translation of the alkali atoms (see Fig. 3**c**). In our analysis, the four different CDW bond order patterns $\{(\mathcal{O}_1, \mathcal{O}_2, \mathcal{O}_3), -(\mathcal{O}_1, -\mathcal{O}_2, \mathcal{O}_3), i(\mathcal{O}_1, -\mathcal{O}_2, \mathcal{O}_3), -i(\mathcal{O}_1, \mathcal{O}_2, \mathcal{O}_3)\}$ are tested where

$$\mathcal{O}_1 = (-\phi_1 I_2 + \phi_1^* \sigma_x) \otimes \sigma_z,\ \mathcal{O}_2 = \sigma_z \otimes (-\phi_2 I_2 + \phi_2^* \sigma_x),\ \mathcal{O}_3 = \phi_3 \sigma_y \otimes \sigma_y + \phi_3^* \sigma_z \otimes \sigma_z. \tag{13}$$

The former/latter two correspond to the TRS/TRSB cases. The sign of order parameter Φ distinguishes two different phases, star of David phase (SD;$\Phi > 0$) and inverse star of David phase (ISD;$\Phi < 0$), for the TRS case. However, the sign is irrelevant for the TRSB case since reversing the sign just connects a given state and its time-reversal partner. To sum up, six different CDW configurations (SD-1/2, ISD-1/2, and TRSB-1/2) are possible and

their symmetry properties are summarized in Table S3.

We now evaluate the ground state of the mean-field Hamiltonian, $H_{\mathrm{MF}}[\Phi] = H_0 + \delta H_{\mathrm{MF}}[\Phi]$. Introducing the unitary transformation,$\tilde{\Psi}_{\boldsymbol{k}} = \mathcal{U}(\boldsymbol{k})\overline{\Psi}_{\boldsymbol{k}}$, the Hamiltonian is

$$H_{\mathrm{MF}}[\Phi] = \sum_{\boldsymbol{k},\alpha_i,\sigma} E_{\mathrm{MF}}^{\alpha_i}[\Phi;\boldsymbol{k}]\,\overline{\Psi}^{\dagger}_{\boldsymbol{k},\alpha_i,\sigma}\overline{\Psi}_{\boldsymbol{k},\alpha_i,\sigma}, \tag{14}$$

where $E_{\mathrm{MF}}^{\alpha_i}[\Phi;\boldsymbol{k}]$ is an α_i-th eigenenergy. Note that the state with tilde/overline is for the basis before/after the Hamiltonian diagonalization. Applying the quasiparticle creation operators $\overline{\Psi}^{\dagger}_{\boldsymbol{k},\alpha_i,\sigma}$ to the electron vacuum gives the ground state energy,

$$|G;\Phi\rangle = \prod_{\alpha_i}\prod_{\boldsymbol{k}\in M_{\alpha_i}[\Phi]} \overline{\Psi}^{\dagger}_{\boldsymbol{k},\alpha_i,\uparrow}\overline{\Psi}^{\dagger}_{\boldsymbol{k},\alpha_i,\downarrow}|0\rangle, \tag{15}$$

where the momentum product is done over the subspace, $M_{\alpha_i}[\Phi] = \{\forall\boldsymbol{k}\in \mathrm{B.Z.} \mid E_{\mathrm{MF}}^{\alpha_i}[\Phi;\boldsymbol{k}] < 0\}$, of the Brillouin zone (BZ) of 2×2 unit cells (see Fig. S14).

We obtain the energy expectation value by sandwiching the ground state to the Hamiltonian, $E[\Phi] \equiv \langle G;\Phi|H_0 + H_{\mathrm{int}}|G;\Phi\rangle$. The explicit forms of each part are given by

$$\langle G;\Phi|H_0|G;\Phi\rangle = 2\sum_{\boldsymbol{k}} \mathrm{Tr}\left[\mathcal{U}^{\dagger}(\boldsymbol{k})\mathcal{H}_0(\boldsymbol{k})\mathcal{U}(\boldsymbol{k})\Xi(\boldsymbol{k})\right], \tag{16}$$

$$\langle G;\Phi|U_{\mathrm{int}}|G;\Phi\rangle = \frac{U}{N}\sum_{\alpha_i}\left[\sum_{\boldsymbol{k}}[\mathcal{U}(\boldsymbol{k})\Xi(\boldsymbol{k})\mathcal{U}^{\dagger}(\boldsymbol{k})]_{\alpha_i,\alpha_i}\right]^2, \tag{17}$$

$$\begin{aligned}\langle G;\Phi|V_{\mathrm{int}}|G;\Phi\rangle = {}& \frac{4}{N}\sum_{\alpha_i,\beta_j}\mathcal{V}_{\alpha_i,\beta_j}(\mathbf{0})\left[\sum_{\boldsymbol{k}}[\mathcal{U}(\boldsymbol{k})\Xi(\boldsymbol{k})\mathcal{U}^{\dagger}(\boldsymbol{k})]_{\alpha_i,\alpha_i}\right]\left[\sum_{\boldsymbol{k}'}[\mathcal{U}(\boldsymbol{k}')\Xi(\boldsymbol{k}')\mathcal{U}^{\dagger}(\boldsymbol{k}')]_{\beta_j,\beta_j}\right] \\ & - \frac{2}{N}\sum_{\alpha_i,\beta_j}\mathcal{V}_{\alpha_i,\beta_j}(\mathbf{0})\left|\sum_{\boldsymbol{k}} e^{-i\boldsymbol{k}\cdot\boldsymbol{\delta}_{\alpha_i\beta_j}}[\mathcal{U}(\boldsymbol{k})\Xi(\boldsymbol{k})\mathcal{U}^{\dagger}(\boldsymbol{k})]_{\alpha_i,\beta_j}\right|^2,\end{aligned} \tag{18}$$

where the direct and exchange terms are contributed as illustrated in Fig. S16. Here we define 12×12 diagonal matrix, $\Xi(\boldsymbol{k})_{\alpha_i,\alpha_i} \equiv \theta(-E_{\mathrm{MF}}^{\alpha_i}[\Phi;\boldsymbol{k}])$, with the Heaviside step function $\theta(x)$ and the 12×12 matrix component of the nearest-neighbour interaction after the Fourier

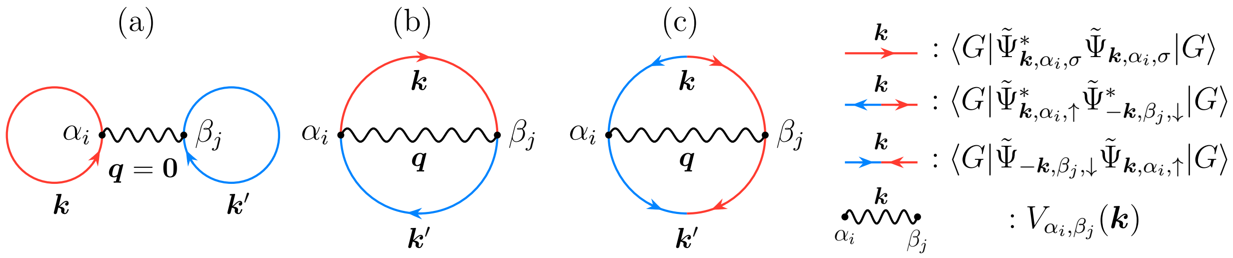

FIG. S16: **Three possible processes contributing to $\langle G|H_{\rm int}|G\rangle$.** The first two processes (a-b) are the conventional direct and exchange process of the four-fermion interactions [10], while the final pairing process (c) is only included for superconducting ground states. Different color denotes the sublattice index (α_i) and detailed diagrammatic rules are explained in the right panel.

transformation $\mathcal{V}_{\alpha_i,\beta_j}$,

$$\begin{aligned}\mathcal{V}(\boldsymbol{q}) \;=\; & V\,[c_1\lambda_1\otimes(I_2+\sigma_x)\otimes I_2+s_1\lambda_4\otimes(-I_2+\sigma_x)\otimes I_2\\ & +c_2\lambda_2\otimes I_2\otimes(I_2+\sigma_x)\otimes s_2\lambda_5\otimes I_2\otimes(-I_2+\sigma_x)\\ & +c_3\lambda_3(I_2\otimes I_2+\sigma_x\otimes\sigma_x)+s_3\lambda_6(I_2\otimes I_2-\sigma_x\otimes\sigma_x)\,]\,.\end{aligned} \tag{19}$$

The matrix satisfies $\mathcal{V}_{\alpha_i,\beta_j}(\boldsymbol{q}) = Ve^{i\boldsymbol{q}\cdot(\boldsymbol{r}_{\beta_j}-\boldsymbol{r}_{\alpha_i})}[\mathcal{V}_{\alpha_i,\beta_j}(\mathbf{0})]$ with $\mathcal{V}(\mathbf{0}) = 2[L_1^+ + L_2^+ + L_3^+]$.

TABLE S3: **CDW orders of monolayer AV$_3$Sb$_5$ considered in the mean-field analysis.** Both time-reversal symmetric and broken types of CDWs are considered. Translational symmetry-lowering effect in monolayer AV$_3$Sb$_5$ plays a crucial role in classifications of order parameters, splitting out a degenerate doublet, CDW-1/2, for each CDW order parameter. The CDW-1 and CDW-2 are connected to each other by a $\sqrt{3}\times 1$ translation of the position of alkali metals.

Time-reversal symmetry	Bond matrix $O_a(\boldsymbol{k})$	Label	Subgroup
TRS	$(\mathcal{O}_1,\mathcal{O}_2,\mathcal{O}_3)$	ISD-1 ($\Phi<0$) or SD-1 ($\Phi>0$)	D_{2h}
	$(-\mathcal{O}_1,\mathcal{O}_2,-\mathcal{O}_3)$	ISD-2 ($\Phi<0$) or SD-2 ($\Phi>0$)	
TRSB	$i(\mathcal{O}_1,-\mathcal{O}_2,\mathcal{O}_3)$	TRSB-1 ($\Phi\neq 0$)	C_{2h}
	$-i(\mathcal{O}_1,\mathcal{O}_2,\mathcal{O}_3)$	TRSB-2 ($\Phi\neq 0$)	

8.3. Superconductivity (SC)

This section provides details on the SC order parameters and their mean-field theories. Introducing the Nambu basis $\xi^T_{\boldsymbol{k}} = (\tilde{\Psi}^T_{\boldsymbol{k}\uparrow}, \tilde{\Psi}^\dagger_{-\boldsymbol{k}\downarrow})$, the mean-field Hamiltonian, or equivalently BdG Hamiltonian, may be written in the 2×2 unit cell as

$$H_{\rm MF}[\Delta] \;=\; \sum_{\boldsymbol{k}} \xi^\dagger_{\boldsymbol{k}} \mathcal{H}_{\rm BdG}(\boldsymbol{k}) \xi_{\boldsymbol{k}}, \quad \mathcal{H}_{\rm BdG}[\Delta;\boldsymbol{k}] = \begin{pmatrix} \mathcal{H}_0(\boldsymbol{k}) & \Gamma(\Delta;\boldsymbol{k}) \\ \Gamma^\dagger(\Delta;\boldsymbol{k}) & -\mathcal{H}^T_0(-\boldsymbol{k}) \end{pmatrix}, \tag{20}$$

where only the spin-singlet pairing channels $\Gamma(\boldsymbol{k})$ with pairing amplitude Δ are considered.

If the pairing gap functions are pinned down, evaluating the ground state of the mean-field Hamiltonian is straightforward. Upon the Bogoliubov transformation with a 24×24 unitary matrix $\mathcal{U}(\boldsymbol{k})$,

$$\xi_{\boldsymbol{k}} = \mathcal{U}(\boldsymbol{k}) \overline{\xi}_{\boldsymbol{k}}, \quad \mathcal{U}(\boldsymbol{k}) = \begin{pmatrix} \mathcal{U}_{pp} & \mathcal{U}_{ph} \\ \mathcal{U}_{hp} & \mathcal{U}_{hh} \end{pmatrix}, \tag{21}$$

we arrive at the mean-field Hamiltonian, $H_{\rm MF}[\Delta]$,

$$H_{\rm MF}[\Delta] = \sum_{\boldsymbol{k},\alpha_i,\sigma} E^{\alpha_i}_{\rm MF}[\Delta;\boldsymbol{k}] \; \left[\overline{\Psi}^\dagger_{\alpha_i,\boldsymbol{k},\uparrow} \overline{\Psi}_{\alpha_i,\boldsymbol{k},\uparrow} - \overline{\Psi}_{\alpha_i,-\boldsymbol{k},\downarrow} \overline{\Psi}^\dagger_{\alpha_i,-\boldsymbol{k},\downarrow} \right], \tag{22}$$

where $E^{\alpha_i}_{\rm MF}[\Phi;\boldsymbol{k}]$ is α_i-th eigenenergy of Bogoliubon. The overline notation is used for denoting the basis after the Bogoliubov transformation. Applying the annihilation operator of Bogoliubov quasiparticles $\overline{\Psi}_{\boldsymbol{k},\alpha_i,\sigma}$ to the electron vacuum, the superconducting ground state is constructed as

$$|G;\Delta\rangle = \prod_{\alpha_i} \prod_{\boldsymbol{k}} \overline{\Psi}_{-\boldsymbol{k},\alpha_i\downarrow} \overline{\Psi}_{\boldsymbol{k},\alpha_i\uparrow} |0\rangle, \tag{23}$$

where the momentum product is performed over the BZ of the 2×2 unit cells.

We now determine the possible pairing channels originating from the interactions Eq.(9). Utilizing the Fierz identity, one find that the nine spin-singlet pairing gap functions labeled

by different irreducible representations may be stabilized under the four-fermion interactions,

$$\Gamma[\{\Delta_R\};\boldsymbol{k}] = \sum_{n=1}^{4}\Delta_{A_g}^{(n)}\hat{\Gamma}_{A_g}^{(n)} + \sum_{n=1}^{2}\Delta_{B_{1g}}^{(n)}\hat{\Gamma}_{B_{1g}}^{(n)} + \Delta_{B_{2u}}^{(1)}\hat{\Gamma}_{B_{2u}}^{(1)} + \sum_{n=1}^{2}\Delta_{B_{3u}}^{(n)}\hat{\Gamma}_{B_{3u}}^{(n)}, \tag{24}$$

where the real order parameter $\Delta_R^{(n)}$ is associated with an irreducible representation R. In Table S4, we tabulate all pairing gap functions $\hat{\Gamma}_R^{(n)}$ written in the 1×1 and 2×2 unit cells.

We now evaluate the energy expectation value $E[\Delta] \equiv \langle G;\Delta|H_0 + H_{\rm int}|G;\Delta\rangle$ from the ground state which yields the final results,

$$\langle G;\Delta|H_0|G;\Delta\rangle = \sum_{\boldsymbol{k}} \mathrm{Tr}\Big(\mathcal{U}_{ph}^\dagger(\boldsymbol{k})\mathcal{H}_0(\boldsymbol{k})\mathcal{U}_{ph}(\boldsymbol{k}) - \mathcal{U}_{hh}^\dagger(\boldsymbol{k})\mathcal{H}_0^T(-\boldsymbol{k})\mathcal{U}_{hh}(\boldsymbol{k}) + \mathcal{H}_0(\boldsymbol{k})\Big), \tag{25}$$

$$\langle G;\Delta|U_{\rm int}|G;\Delta\rangle = \frac{U}{N}\sum_{\alpha_i}\Big|\sum_{\boldsymbol{k}}\big[\mathcal{U}_{hh}(\boldsymbol{k})\mathcal{U}_{ph}^\dagger(\boldsymbol{k})\big]_{\alpha_i,\alpha_i}\Big|^2 + \Big[\sum_{\boldsymbol{k}}\big[\mathcal{U}_{ph}(\boldsymbol{k})\mathcal{U}_{ph}^\dagger(\boldsymbol{k})\big]_{\alpha_i,\alpha_i}\Big]^2, \tag{26}$$

$$\begin{aligned}\langle G;\Delta|V_{\rm int}|G;\Delta\rangle &= \frac{2}{N}\sum_{\alpha_i,\beta_j}\mathcal{V}_{\alpha_i,\beta_j}(\mathbf{0})\Big|\sum_{\boldsymbol{k}} e^{-i\boldsymbol{k}\cdot\boldsymbol{\delta}_{\alpha_i\beta_j}}[\mathcal{U}_{hh}(\boldsymbol{k})\mathcal{U}_{ph}(\boldsymbol{k})^\dagger]_{\alpha_i\beta_j}\Big|^2 \\ &+ \frac{4}{N}\sum_{\alpha_i,\beta_j}\mathcal{V}_{\alpha_i,\beta_j}(\mathbf{0})\big[\sum_{\boldsymbol{k}}\mathcal{U}_{ph}(\boldsymbol{k})\mathcal{U}_{ph}(\boldsymbol{k})^\dagger\big]_{\alpha_i\alpha_i}\big[\sum_{\boldsymbol{k}'}\mathcal{U}_{ph}(\boldsymbol{k}')\mathcal{U}_{ph}(\boldsymbol{k}')^\dagger\big]_{\beta_j\beta_j} \\ &- \frac{2}{N}\sum_{\alpha_i,\beta_j}\mathcal{V}_{\alpha_i,\beta_j}(\mathbf{0})\Big|\sum_{\boldsymbol{k}} e^{-i\boldsymbol{k}\cdot\boldsymbol{\delta}_{\alpha_i\beta_j}}[\mathcal{U}_{ph}(\boldsymbol{k})\mathcal{U}_{ph}(\boldsymbol{k})^\dagger]_{\alpha_i\beta_j}\Big|^2.\end{aligned} \tag{27}$$

Note that three possible fully contracted terms contribute to the ground state energy of the superconducting state and their diagrammatic interpretation is expressed in Fig. S16.

TABLE S4: **Nine SC orders of monolayer AV_3Sb_5 are considered in the mean-field analysis.** Spin-singlet superconducting pairings are classified by irreducible representations (R) of the point group D_{2h}. Condensation of $\hat{\Gamma}_R$ channels can lower the interaction Hamiltonian energy, which preserves $\mathcal{T}_{1\times 1}$ symmetry. The momentum dependence of pairing gap function is encoded in $(c_i, s_i) \equiv (\cos \boldsymbol{k}\cdot\boldsymbol{R}_i, \sin \boldsymbol{k}\cdot\boldsymbol{R}_i)$. To express pairing functions in both 1×1 and 2×2 unit cells, the 3×3 Gell Mann matrice λ_a and the 12×12 matrices $L_a^{\pm,0} = \lambda_a \otimes M_a^{\pm,0}$ are introduced with 4×4 matrices $M_a^{\pm,0}$. The lowest order of basis functions associated with representation R are provided in the fifth column.

R	Pairing in 1×1 unit cell	Pairing in 2×2 unit cell $\hat{\Gamma}_R(\boldsymbol{k})$	Label	Basis function
A_g	$\frac{1}{\sqrt{3}}\lambda_0, \frac{1}{\sqrt{2}}\left[\frac{\sqrt{3}}{2}\lambda_7 + \frac{1}{2}\lambda_8\right],$ $\frac{1}{\sqrt{2}}[c_1\lambda_1 + c_2\lambda_2],$ $c_3\lambda_3$	$\frac{1}{\sqrt{3}}L_0^0, \frac{1}{\sqrt{2}}\left[\frac{\sqrt{3}}{2}L_7^0 + \frac{1}{2}L_8^0\right],$ $\frac{1}{\sqrt{2}}\left[c_1L_1^+ + c_2L_2^+ - (s_1L_4^- + s_2L_5^-)\right],$ $\left[c_3L_3^+ - s_3L_6^-\right]$	$\Gamma^{(1)}_{A_g}, \Gamma^{(2)}_{A_g},$ $\Gamma^{(3)}_{A_g},$ $\Gamma^{(4)}_{A_g}$	x^2, y^2, z^2
B_{1g}	$\frac{1}{\sqrt{2}}\left[-\frac{1}{2}\lambda_7 + \frac{\sqrt{3}}{2}\lambda_8\right],$ $\frac{1}{\sqrt{2}}[c_1\lambda_1 - c_2\lambda_2]$	$\frac{1}{\sqrt{2}}\left[-\frac{1}{2}L_7^0 + \frac{\sqrt{3}}{2}L_8^0\right],$ $\frac{1}{\sqrt{2}}\left[c_1L_1^+ - c_2L_2^+ - (s_1L_4^- - s_2L_5^-)\right]$	$\Gamma^{(1)}_{B_{1g}},$ $\Gamma^{(2)}_{B_{1g}}$	xy
B_{2u}	$\frac{1}{\sqrt{2}}[s_1\lambda_4 - s_2\lambda_5]$	$\frac{1}{\sqrt{2}}\left[s_1L_4^+ - s_2L_5^+ - (c_1L_1^- - c_2L_2^-)\right]$	$\Gamma^{(1)}_{B_{2u}}$	y
B_{3u}	$\frac{1}{\sqrt{2}}[s_1\lambda_4 + s_2\lambda_5],$ $s_3\lambda_6$	$\frac{1}{\sqrt{2}}\left[s_1L_4^+ + s_2L_5^+ + (c_1L_1^- + c_2L_2^-)\right],$ $\left[s_3L_6^+ - c_3L_3^-\right]$	$\Gamma^{(1)}_{B_{3u}},$ $\Gamma^{(2)}_{B_{3u}}$	x

8.4. Details of variational method

In this section, we show that our variational method is essentially equivalent to solving a self-consistency equation at zero temperature. The self-consistency equation is obtained by differentiating free energy with respect to the mean-field order parameter to find a stationary point. On the other hand, the variational method directly determines the minimum of the free energy by varying the order parameter of the wave function. Therefore, the equivalence between the two methods is well established, unless the solution is located at local minimum points. To check the equivalence, we plot the magnitudes of the order parameter carried out through the two methods in Fig. S17, where the perfect matching between the two methods is demonstrated.

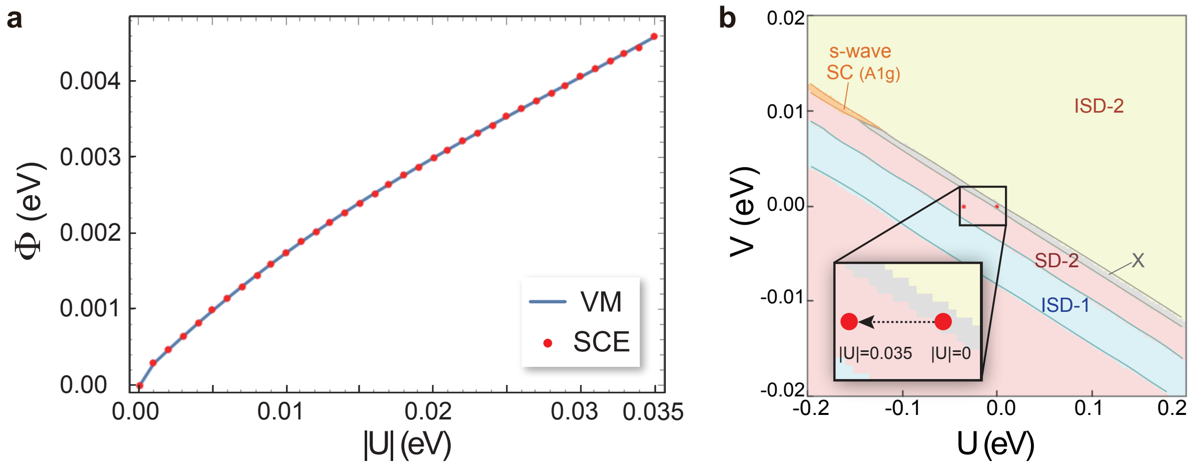

FIG. S17: **Order parameter (SD-2) amplitude of phase as a function of U and the corresponding phase diagram at the chemical potential $\mu = -6$meV. a.** Order parameter amplitude is obtained by solving the self-consistency equation (SCE) and variational method (VM) at zero temperature, which gives equivalent results ($U < 0, V = 0$) **b.** The mean-field phase diagram at the chemical potential $\mu = -6$meV. In particular interest phase space considered in (a), the SD-2 CDW order is stabilized (Inset).

8.5. Comment on the attractive interaction

In this section, we would like to add a few words on the effect of the phonons in the mean-field phase diagram. As well established in the famous BCS theory, the phonon-electron coupling induces an effective attractive interaction after the renormalization. For

example, when the phonon is integrated out, the electron-electron interaction is written as,

$$W_{\text{eff}}(\boldsymbol{q}, \omega) = W_0(\boldsymbol{q}) \frac{\omega^2}{\omega^2 - \omega_D^2}, \tag{28}$$

with bare interaction $V_0(\boldsymbol{q})$, and Debye frequency ω_D. It is clear that the sign can be reversed in the low-frequency limit, $\omega < \omega_D$. In this analogy, the attractive values of (U, V) are taken into account for the mean-field calculations.

Supplementary Note 9. Details on mean-field phase diagram

We here show the phase diagrams in the range of chemical potential $\mu = -10 \sim 30$meV to elucidate the effect of fillings on competing orders of the monolayer AV_3Sb_5, while only the two representative cases are provided in the main text. Our zero-temperature mean-field theory is performed in the same manner by adopting the same tight-binding (TB) and extended Hubbard model explained in the previous section.

Figure S18**a** illustrates DFT and TB band structures where the TB parameters are set as $(\epsilon, t, t_2, \delta\epsilon, \delta t) = (0.01, 0.42, 0.03, -0.033, 0.01)$. Figure S18**b** shows density of states indicating the two different types of VHSs. Figures S18**c-g** display the mean-field phase diagrams in (U, V) space at $\mu = -10, -6, 9, 20$, and 30 meV, where the six CDW configurations and nine SC orders in Table S3 and S4 are considered. A few remarks are as follows. First, the phase diagrams are significantly changed and a variety of exotic phases can be achieved as a function of the chemical potential. Second, the electronic instabilities associated with the type-I and II VHSs are quite distinct. While the SD phases dominate at type-I VHS filling (Fig. S18**e**), both ISD and SD phases are stabilized in the wide range of (U, V) at type-II VHS filling (Fig. S18**d**). The s-wave SC ($\Gamma^{(1)}_{A_g}$) appears for negative U at both filings, but d-wave SC ($\Gamma^{(1)}_{B_{1g}}$) is only shown at type-I VHS filling. Third, the TRSB CDW phase appears when the chemical potential is shifted above both VHS points (Fig. S18**g**). The topological property of the TRSB CDW phase manifests in nonzero Chern number and subsequent anomalous Hall conductivity (see Fig. 5**a,b**).

We next pin down the stoichiometry-enforced symmetry lowering effects of the monolayer system $(\mathcal{T}_{\sqrt{3}\times 1}, D_{2h})$ on the phase diagrams, by comparing with the phase diagrams obtained for a structure having $\mathcal{T}_{1\times 1}$ and D_{6h} symmetries. Note that $\mathcal{T}_{1\times 1}$ and D_{6h} symmetries are the same as the symmetries of the bulk system (Fig. 1**a**). For the $\mathcal{T}_{1\times 1}$ and D_{6h} symmetries, CDW and SC order parameters are newly classified in Table S5 and S6. The key difference of $(\mathcal{T}_{1\times 1}, D_{6h})$ symmetric system is that the two distinct states CDW-1 and CDW-2 in the presence of $(\mathcal{T}_{\sqrt{3}\times 1}, D_{2h})$ symmetry now become equivalent due to the restored translational symmetry. Figures S19**a-b** show DFT and TB band structures and density of states of the D_{6h} system where the TB parameters are set as $(\epsilon, t, t_2, \delta\epsilon, \delta t) = (0.01, 0.42, 0.03, 0, 0)$. Figures S19**c-g** display the mean-field phase diagrams at $\mu = -10, 0, 10, 20$, and 30 meV. Our analysis makes the following observation. First, the superconducting phase in the

D_{2h} system is less favored compared to the D_{6h} system, which is clearly seen in the phase diagrams at the type-I VHS filling of the two systems (Figs. S18**e** and S19**e**). Interestingly, a spin-singlet f-wave SC ($\Gamma_{B_{2u}}$) is found below the VHS filling for the D_{6h} system ($\mu = -10$ meV). We note that the emergence of the spin-triplet f-wave SC is reported in the previous theoretical study for the bulk system [7]. Second, the TRSB CDW has stabilized in a wide range of chemical potential for the D_{6h} system compared to the D_{2h} system. The occurrence of the TRSB CDW is consistent with the various experiments [11–14] observing TRSB CDW.

TABLE S5: **CDW orders in the D_{6h} symmetry considered in the mean-field analysis.** Both time-reversal symmetric and broken types of CDWs are considered. Two distinct states in the CDW doublet, (CDW-1/2), in the $\mathcal{T}_{\sqrt{3}\times 1}$ and D_{2h} symmetries are now equivalent in the $\mathcal{T}_{1\times 1}$ and D_{6h} symmetries.

Time-reversal symmetry	Bond matrix $O_a(\boldsymbol{k})$	Label	Subgroup
TRS	$(\mathcal{O}_1, \mathcal{O}_2, \mathcal{O}_3)$	ISD ($\Phi < 0$) or SD ($\Phi > 0$)	D_{6h}
TRSB	$-i(\mathcal{O}_1, \mathcal{O}_2, \mathcal{O}_3)$	TRSB ($\Phi \neq 0$)	C_{6h}

TABLE S6: **Three SC orders in the D_{6h} symmetry are considered in the mean-field analysis.** One-dimensional spin-singlet superconducting pairings are classified by the irreducible representations (R) of the point group D_{6h}. The momentum dependence of pairing gap function is encoded in $(c_i, s_i) \equiv (\cos \boldsymbol{k}\cdot\boldsymbol{R}_i, \sin \boldsymbol{k}\cdot\boldsymbol{R}_i)$ with $\omega \equiv e^{\frac{2\pi}{3}i}$. The lowest order of basis functions associated with representation R is provided in the fifth column.

R	Pairing in 1 × 1 unit cell	Pairing in 2 × 2 unit cell $\Gamma_R(\boldsymbol{k})$	Label	Basis function
A_{1g}	$\frac{1}{\sqrt{3}}\lambda_0$,<br>$\frac{1}{\sqrt{3}}\left[c_1\lambda_1 + c_2\lambda_2 + c_3\lambda_3\right]$	$\frac{1}{\sqrt{3}}L_0^0$,<br>$\frac{1}{\sqrt{3}}\left[c_1L_1^+ + c_2L_2^+ + c_3L_3^+ - (s_1L_4^- + s_2L_5^- + s_3L_6^-)\right]$	$\Gamma^{(1)}_{A_{1g}}$,<br>$\Gamma^{(2)}_{A_{1g}}$	x^2+y^2, z^2
B_{2u}	$\frac{1}{\sqrt{3}}\left[s_1\lambda_4 + s_2\lambda_5 - s_3\lambda_6\right]$	$\frac{1}{\sqrt{3}}\left[s_1L_4^+ + s_2L_5^+ - s_3L_6^+ - (c_1L_4^- + c_2L_5^- - c_3L_6^-)\right]$	$\Gamma^{(1)}_{B_{2u}}$	$x(x^2-3y^2)$

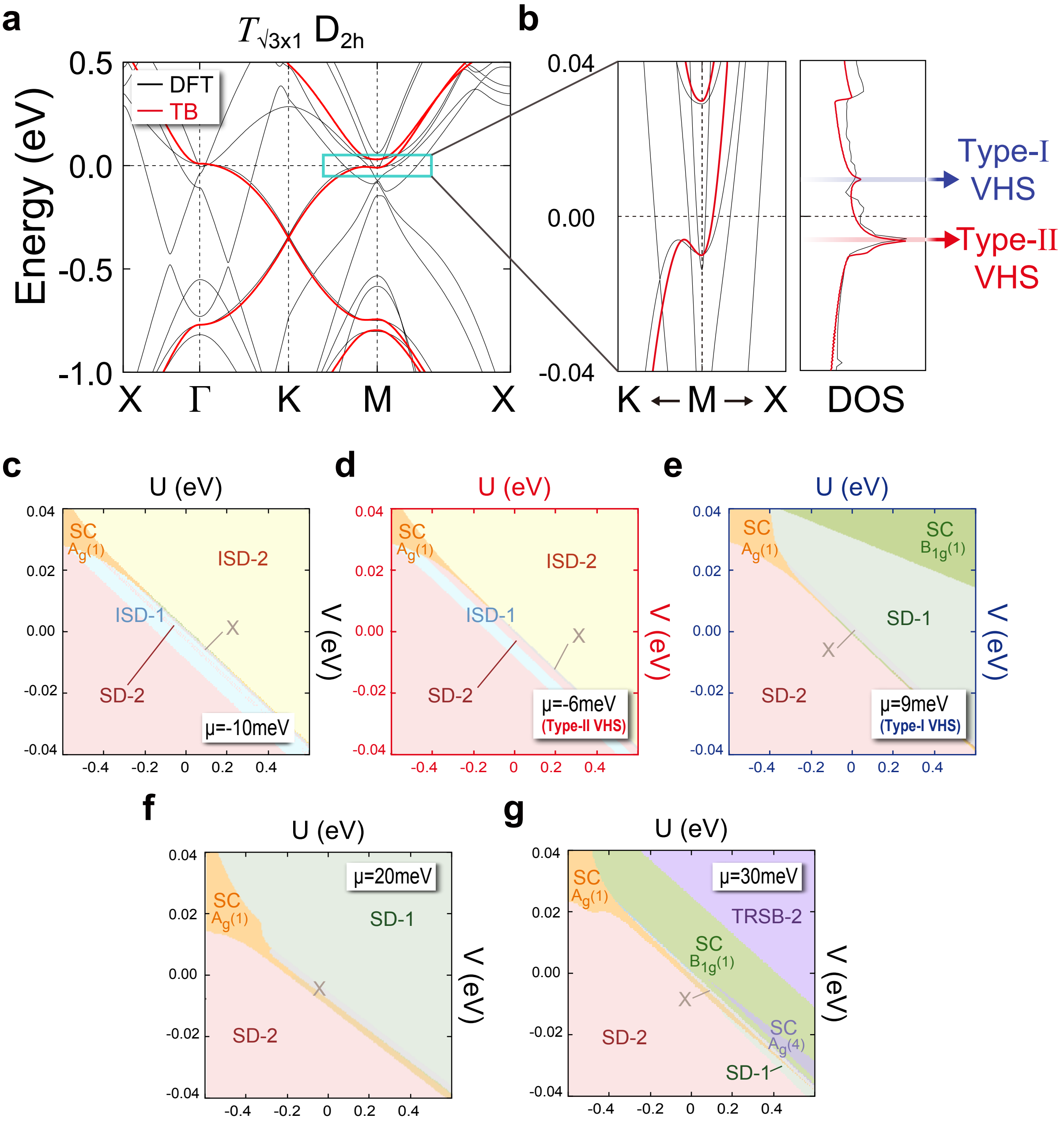

FIG. S18: **Electronic structures and chemical potential dependence of phase diagram of the monolayer AV_3Sb_5.** **a-b** DFT and TB band structures of monolayer KV_3Sb_5, denoted by black and red color. Density of states and the position of type-I/II VHSs are illustrated. **c-g** Mean-field phase diagram at the chemical potentials $\mu = -10, -6, 9, 20,$ and 30 meV. For numerical evaluations, we set the tight-binding parameters $(\epsilon, t, t_2, \delta\epsilon, \delta t) = (0.01, 0.42, 0.03, -0.033, 0.01)$. Emergence and competition of various CDW and superconducting phases are highly contingent upon the fillings. The CDW and superconducting orders considered in the mean-field theory are tabulated in Tables S3 and S4. For numerical evaluations, we obtain 161 data points $\phi_i \in [-0.08, 0.08]$ of the ground state energy, with 80×80 k-points.

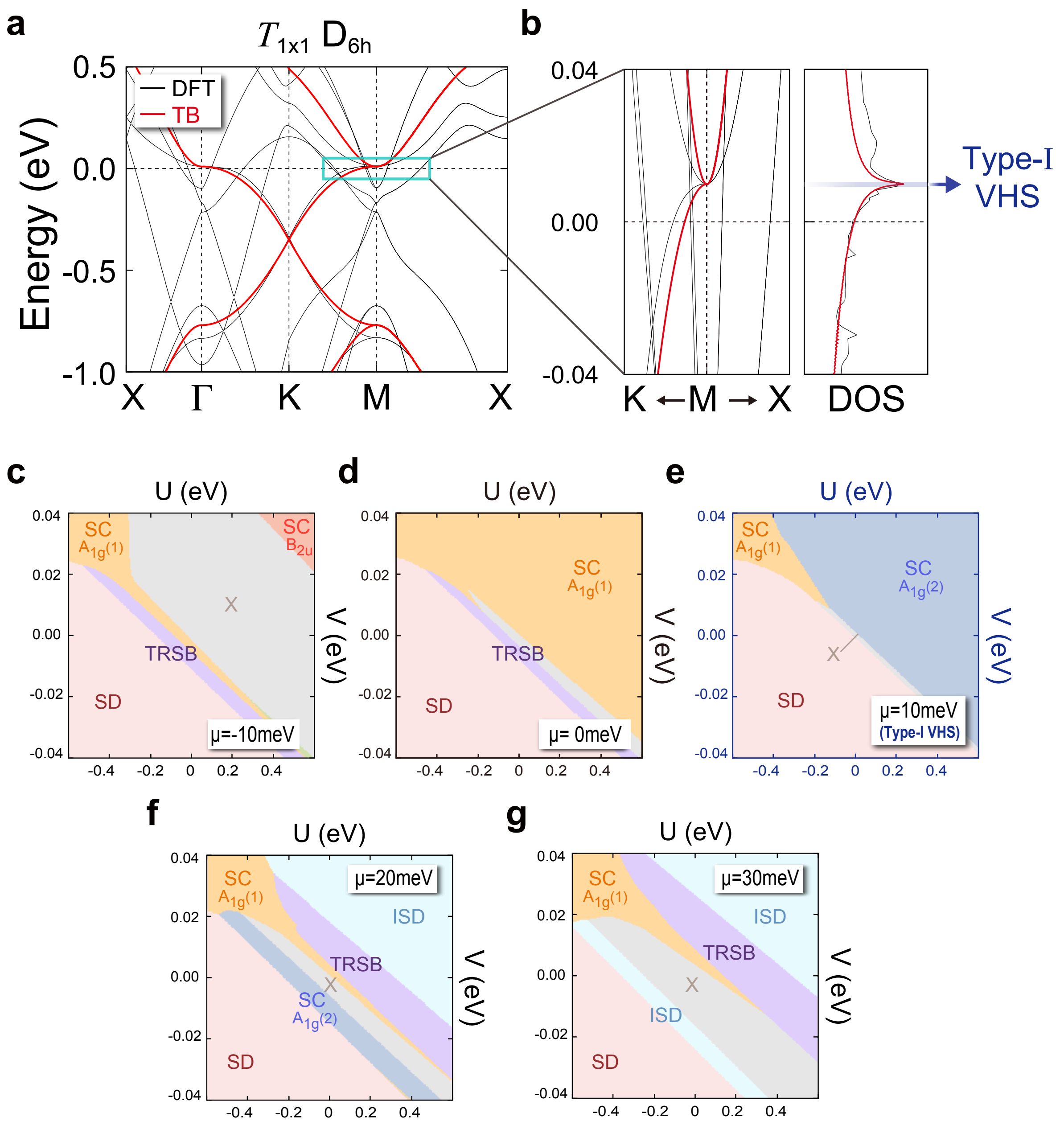

FIG. S19: **Electronic structures and chemical potential dependence of phase diagram of AV_3Sb_5 with $\mathcal{T}_{1\times1}$ and D_{6h} symmetries. a-b** DFT and TB band structures of KV_3Sb_5 are illustrated by black and red colors. Here, although the structure with D_{6h} point group symmetry has $\mathcal{T}_{1\times1}$ translational symmetry, we plot the band structures along the BZ of the $\sqrt{3}\times1$ unit cell to compare with the structure having $\mathcal{T}_{\sqrt{3}\times1}$ and D_{2h} symmetries in Fig. S18. The density of states and the position of type-I VHS are expressed in **b**. **c-g** Mean field phase diagram at the chemical potentials $\mu = -10, 0, 10, 20,$ and 30 meV. We set the tight-binding parameters $(\epsilon, t, t_2, \delta\epsilon, \delta t) = (0.01, 0.42, 0.03, 0, 0)$. Emergence and competition of various CDW and superconducting phases are highly contingent upon the fillings. The CDW and superconducting orders considered in the mean-field theory are tabulated in Tables S5 and S6. For numerical evaluations, we obtain 161 data points $\phi_i \in [-0.08, 0.08]$ of the ground state energy, with 80×80 k-points.

Supplementary Note 10. Estimation of U and V values and their tunability

We evaluate the (U, V) values in monolayer by using the first-principles constrained random phase approximation (cRPA) method [15]. In cRPA method, we divide the full polarization function into two parts as $P = P_t + P_r$ in which P_t and P_r are calculated within the target and rest orbitals, respectively. The effective Coulomb interaction matrix $\mathcal{U}$ is computed via $\mathcal{U} = [1 - \mathcal{U}_{\text{bare}} P_r]^{-1} \mathcal{U}_{\text{bare}}$ where $\mathcal{U}_{\text{bare}}$ is a bare Coulomb interaction. We then average the matrix $\mathcal{U}$ to obtain U, V parameters. We find that our screened Hubbard parameters U and V in the bulk are quite similar to those of the previous study [16], as shown in Table S7. The values of U and V in the monolayer are quite comparable to those of bulk. The effective U and V values depend on the target orbital models because the rest orbitals screen the target orbitals. Interestingly, due to the large screening effect, the U and V values are strongly renormalized as $U = 0.58$ and $V = 0.07$ eV for our A_g orbital TB model used for mean-field calculations. The estimated U and V values are comparable to the U and V ranges considered in our mean-field phase diagram. We note that the effective U and V values obtained from the cRPA calculations can be changed and even become negative if the electron-phonon coupling is considered as we discussed in section 7.5.

TABLE S7: **On-site U and nearest-neighbor V Hubbard interaction parameters calculated by cRPA method [15] with the weighting approach [17].** Our A_g orbital TB model used for mean-field calculations includes three V-d target orbitals of $d_{3z^2-r^2}$, $d_{x^2-y^2}$, and d_{xy} in global coordinates (Fig. S15), dubbed V-$d3$ model.

	Monolayer		Bulk	
Target orbitals	$U(F_0)$ (eV)	V (eV)	$U(F_0)$ (eV)	V (eV)
All (bare)	14.20	3.93	15.79	4.46
V-d Sb-p	5.87	1.96	5.34	1.48
V-d	0.77	0.11	0.80	0.08
V-$d3$ (A_g orbital)	0.58	0.07	0.60	0.06

We also show that the U and V values are tunable under mechanical strains, which allows us to access diverse phases. As shown in Fig. S20**a**, the calculations reveal that the U value changes from 0.55 eV to 0.67 eV from 0 to 4 percents uniform tensile strains. Similarly, the V value from 0.07 eV to 0.11 eV under the same modulation of tensile strain. Encouragingly,

within these changes of U and V values, we find that diverse phases are accessible. For example, for the chemical potential $\mu = 10$ meV, two different superconducting phases with d-wave (B_{1g}) and p-wave (B_{2u}) symmetries are accessible as shown in Fig. S20**b**. In addition, for the chemical potentials $\mu = 20$ and 30 meV, the SD-1 and TRSB-2 CDW and superconducting phases are observable within the ranges of the U and V values as shown in Figs. S20**c-d**. Therefore, we believe that diverse phases can be accessed in experiments via strain engineering.

We expect that another feasible route to tune the U and V values is to utilize different substrates, gate dielectrics, or gate configurations, which effectively change the screening of Coulomb interaction as in many two-dimensional systems [18–20].

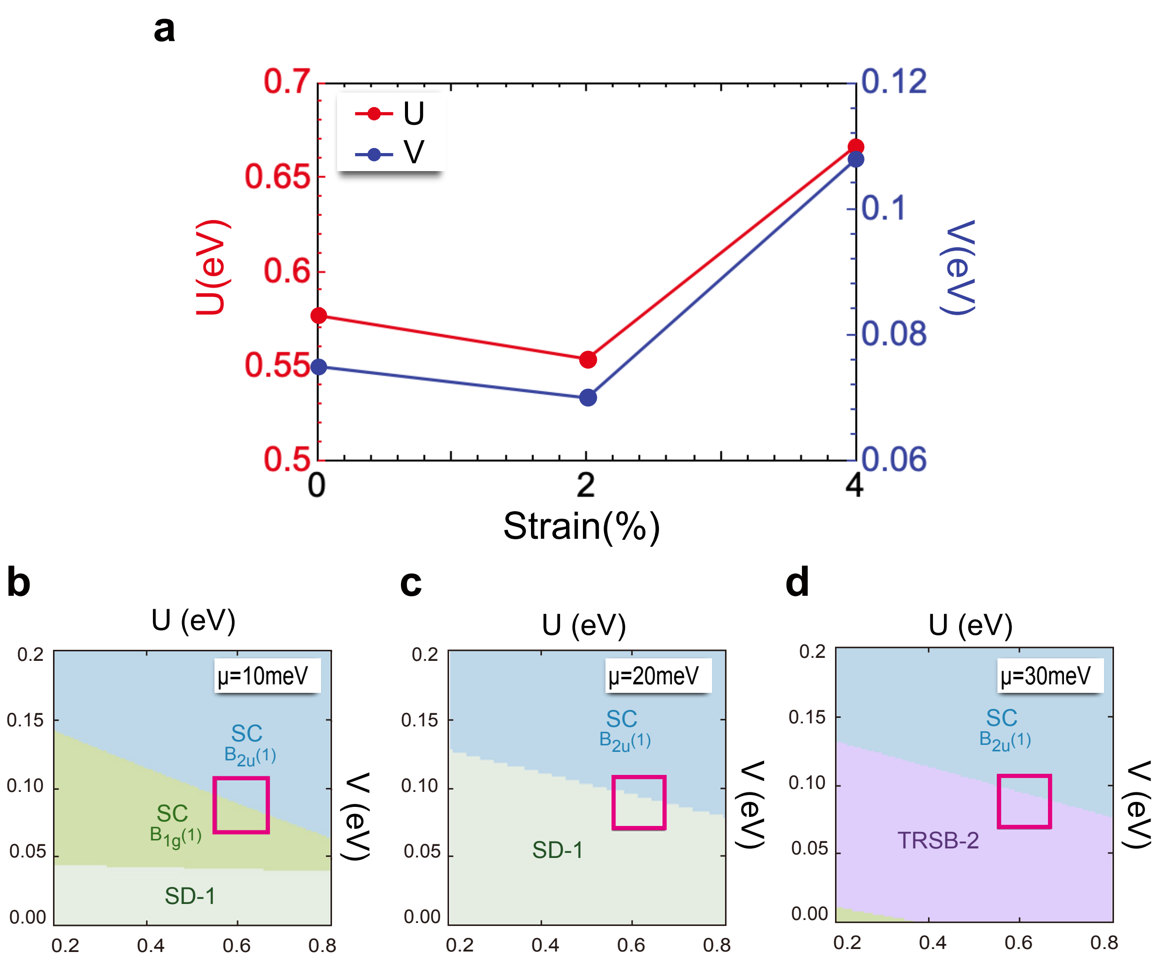

FIG. S20: **a** U and V values as a function of uniform tensile strain. **b-d** The phase diagrams in U and V space for various chemical potentials. **b** $\mu = 10$ meV **c** $\mu = 20$ meV, and **d** $\mu = 30$ meV. The red boxed regions show the accessible phases under strain engineering.

Supplementary Note 11. Comments on Incommensurate CDW

In this section, we provide some discussion about incommensurate CDW (IC-CDW) orders, which did not mainly cover in the main text. It is generally known that the incommensurate CDW (IC-CDW) orders are much more vulnerable under thermal fluctuations and impurities [21]. Within an IC-CDW state, all positions of charge density relative to the crystal lattice are equally likely, in stark contrast to the commensurate CDW phase. Applying this perspective to the monolayer AV_3Sb_5 system, the bond orders with any phase, $\phi_{\alpha\beta}$, at given incommensurate Q vector, $\boldsymbol{Q}_{\alpha\beta}$, are basically degenerate, where

$$\Phi_{\alpha\beta} = \sum_{\boldsymbol{R}} \left[\cos(\boldsymbol{Q}^{IC}_{\alpha\beta} \cdot \boldsymbol{R} + \phi_{\alpha\beta}) \langle \alpha^\dagger_{\boldsymbol{R}} \beta_{\boldsymbol{R}} \rangle + \text{C.C.} \right], \tag{29}$$

with sublattice index $(\alpha, \beta) \in \text{AB}, \text{BC}, \text{CA}$. We note that the gapless goldstone, namely phason, can be carried by the phase of complex CDW order, $\phi_{\alpha\beta}$. Then, the dimensionality plays a key role since the continuous symmetry associated with IC-CDW cannot be broken in a dimension lower than the critical dimension, $d_{l,c} = 2$, at finite temperature, due to the celebrated Mermin-Wagner theorem.

To resolve the IC-CDW issue more concretely, we additionally performed numerical calculations, employing one of the well-known numerical approximation methods to incorporate incommensurate CDW orders [22]. We obtain a better picture of the clean monolayer kagome metals at zero temperature, finding the intriguing competing physics between IC-CDW and 2×2 CDW, as summarized in Fig.S21. Our new results can be considered as a demonstration of the new physics originating from type-II VHS points (P_i) which are off high-symmetry momenta (M_i). Below, we briefly sketch how the incommensurate CDW orders are considered.

In our improved mean-field ansatz, all IC-CDW configurations are included by extending the original ansatz which is restricted to access 2×2 CDW orders only. The possible Q vectors of IC-CDW orders associated with (AB, BC, CA) bonds are constructed by connecting the rearranged VHS points,

$$\boldsymbol{Q}^{IC}_{i,j} = (\boldsymbol{P}^{(2)}_i - \boldsymbol{M}_1, \boldsymbol{P}^{(3)}_j - \boldsymbol{P}^{(2)}_i, \boldsymbol{M}_1 - \boldsymbol{P}^{(3)}_j), \tag{30}$$

where $P_i^{(n)}$ is a shifted type-II VHS point from M_n by $d\boldsymbol{q}_i$ (See Fig. S22 i,ii of (a-d)). Here, $d\boldsymbol{q}_{1,3} = \mp[0.0498\boldsymbol{b}_1 - 0.0315\boldsymbol{b}_2]$, $d\boldsymbol{q}_{2,4} = \mp[0.315\boldsymbol{b}_1 - 0.0498\boldsymbol{b}_2]$ are defined with a 1×1 reciprocal vector $\boldsymbol{b}_{1,2}$. Depending on choosing different type-II VHSs, $(\boldsymbol{P}_i^{(2)}, \boldsymbol{P}_j^{(3)})$, there are 16 number of cases of IC-CDWs, distinguished by deviated Q vectors, $d\boldsymbol{Q}_{i,j}^{IC}$, from those of 2×2 CDW orders $\boldsymbol{Q}_{2\times2} \equiv (\boldsymbol{M}_2 - \boldsymbol{M}_1, \boldsymbol{M}_3 - \boldsymbol{M}_2, \boldsymbol{M}_1 - \boldsymbol{M}_3)$, whose explicit form is given by

$$d\boldsymbol{Q}_{i,j}^{IC} \equiv \boldsymbol{Q}_{i,j}^{IC} - \boldsymbol{Q}_{2\times2} = (d\boldsymbol{q}_i, d\boldsymbol{q}_j - d\boldsymbol{q}_i, -d\boldsymbol{q}_j). \tag{31}$$

Here, the D_{2h} symmetry simplifies the possible cases into 4 ones ($i = 3, j \in 1, 2, 3, 4$) and the associated possible $3Q$ and $1Q$ IC-CDW orders are taken into account (See Fig.S22). Note that $3Q$ orders mean that the relative amplitudes of (Q_{AB}, Q_{BC}, Q_{CA}) bonds are equivalent, while $1Q$ CDW order has only one finite amplitude of bond order among (Q_{AB}, Q_{BC}, Q_{CA}) bonds.

Technically, we have performed the supercell calculations with commensurate approximations, one of the well-established theoretical methods used for IC-CDW analysis. Following the previous literature [22], we could approximate the Q vector of IC-CDW to the closest rational numbers of the 1×1 reciprocal vector, $\boldsymbol{b}_i$, which reproduce similar physical properties of the quasicrystal. For example, within 20×32 supercell approximation, IC-CDW wave vectors are approximated as

$$d\boldsymbol{q}_{1,3} = \mp[0.0498\boldsymbol{b}_1 - 0.0315\boldsymbol{b}_2] \to d\boldsymbol{q}_{1,3}^{\mathrm{app}} \mp [\frac{1}{20}\boldsymbol{b}_1 - \frac{1}{32}\boldsymbol{b}_2], \tag{32}$$

$$d\boldsymbol{q}_{2,4} = \mp[0.0314\boldsymbol{b}_1 - 0.0498\boldsymbol{b}_2] \to d\boldsymbol{q}_{2,4}^{\mathrm{app}} \mp [\frac{1}{32}\boldsymbol{b}_1 - \frac{1}{20}\boldsymbol{b}_2]. \tag{33}$$

The accuracy of these approximations increases as reducing the difference between actual values ($d\boldsymbol{q}_i$) and approximated ones ($d\boldsymbol{q}_i^{\mathrm{app}}$) by enlarging the supercell size (say, $N_1 \times N_2$), as shown in Fig.S23, where approximation error, $(|d\boldsymbol{q}_i - d\boldsymbol{q}_i^{\mathrm{app}}|/d\boldsymbol{q}_i)$, is plotted as a function of the supercell size, (N_1, N_2). We note that our approximated IC-CDW Hamiltonian is already a 1920×1920 dimension, and increasing the matrix size is technically complicated since both the time and memory cost scale grows quadratically, $\propto (N_1 N_2)^2$ [23].

Based on our 20×32 supercell calculations, we obtained the new phase diagram at $U \neq 0, V = 0$, as depicted in Fig.S21. For numerical integration of k points, we utilized the

61×61 meshes in the reduced Brillouin zone. We found that the incommensurate CDW replaces the 2×2 CDW in the provided phase diagram, but it cannot be an evidence of instability of the commensurate ISD, as the replacement only occurs, as we fine-tunned the chemical potential to the type-II VHS. By contrast, when the chemical potential is shifted, the 2×2 commensurate ISD phase in a wide range of repulsive interactions, suggesting the stability of the 2×2 commensurate ISD phase. To demonstrate this, we showed the phase diagrams at two different fillings $\mu = -5$ meV and $\mu = -7$ meV, respectively. As shown in Fig. S21, the commensurate ISD phase survives the incommensurate CDW at $\mu = -7$ meV in stark contrast to the $\mu = -5$ meV case, where the IC-CDW are energetically favored in the broad region of $U > 0$. We would like to highlight that the 2×2 commensurate CDW phase is as much as accessible as the IC-CDW phase due to the marked tunability of competing orders in the monolayered platform. This feature is also manifested in our susceptibility calculations (Fig. 3, main text), since the position of the peak is highly affected by chemical potentials. The competition between IC-CDW and 2×2 CDW phases is very versatile under varying interaction strength due to the tiny energy differences and more sophisticated analysis with advanced numerical methods is required for investigating these competing physics in the broad ragnge of chemical potentials.

Our new finding shows that IC-CDW orders are relevant in monolayer AV_3Sb_5 due to the symmetry breaking. Interestingly, the competition between 2×2 CDW and IC-CDW is also recently observed in the hole-doped CsV_3Sb_5 bulk samples, which is attributed to the symmetry breaking [24]. In this respect, we believe that our revised manuscript offers timely new physics in the kagome metal community, calling for future sophisticated theoretical and experimental studies to explore the interplay of physics between IC-CDW and other complex orders.

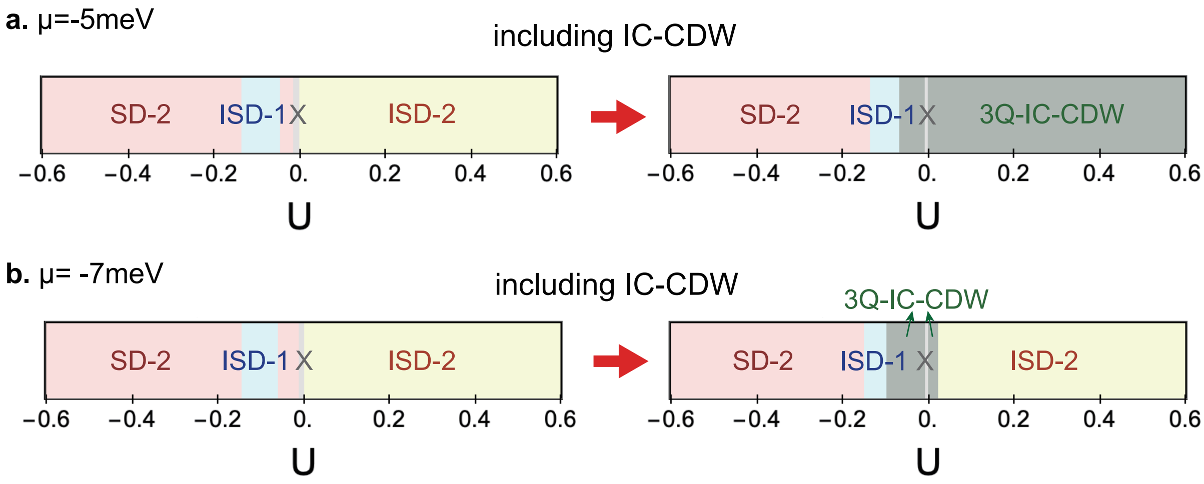

FIG. S21: **Chemical potential dependence of incommensurate CDW phase.** Phase diagrams are calculated at two different fillings (a) $\mu = -5$ meV and (b) $\mu = -7$ meV with (right) and without (left) the incommensurate CDW order. The order parameters of the incommensurate CDWs are given in Fig. S22. These are representative fillings near the type-II filling (μ = -6 meV), which lead to a contrasting impact on the competition between the commensurate and incommensurate CDWs. For repulsive interactions ($U > 0$), the commensurate CDW (ISD-2) dominates over the incommensurate CDW for $\mu = -7$ meV, whereas the incommensurate CDW is dominant for $\mu = -5$ meV. The convergent results are obtained by using 20×32 supercell calculations with 61×61 $\boldsymbol{k}$ mesh points in the reduced Brillouin zone.

TABLE S8: **The shifted Q-vectors of IC-CDW from 2×2 CDW one, $dQ^{IC}_{i,j}$ and their approximated values, $dQ^{IC,\text{app}}_{i,j}$, within 20×32 supercell for four different cases.** The three components in $dQ^{IC}_{i,j}$, and $dQ^{IC\text{app}}_{i,j}$ correspond to the Q-vector of (AB,BC,CA) bonds. The four different cases are classified by choice of different VHS points $(P^{(2)}_i, P^{(3)}_j)$, as illustrated in the Fig.S22.

Case	$d\boldsymbol{Q}^{IC}_{i,j}$	$d\boldsymbol{Q}^{IC,\text{app}}_{i,j}$ within 20×32 supercell
Case 1 $(i,j) = (3,3)$	$[0.0498\boldsymbol{b}_1 - 0.0315\boldsymbol{b}_2, \boldsymbol{0}, -0.0498\boldsymbol{b}_1 + 0.0315\boldsymbol{b}_2]$	$\left[\frac{1}{20}\boldsymbol{b}_1 - \frac{1}{32}\boldsymbol{b}_2, \frac{0}{20}\boldsymbol{b}_1 + \frac{0}{32}\boldsymbol{b}_2, -\frac{1}{20}\boldsymbol{b}_1 + \frac{1}{32}\boldsymbol{b}_2\right]$
Case 2 $(i,j) = (3,4)$	$[0.0498\boldsymbol{b}_1 - 0.0315\boldsymbol{b}_2, -0.0183\boldsymbol{b}_1 - 0.0183\boldsymbol{b}_2, -0.0315\boldsymbol{b}_1 + 0.0498\boldsymbol{b}_2]$	$\left[\frac{1}{20}\boldsymbol{b}_1 - \frac{1}{32}\boldsymbol{b}_2, \frac{0}{20}\boldsymbol{b}_1 + \frac{1}{32}\boldsymbol{b}_2, -\frac{1}{20}\boldsymbol{b}_1 + \frac{2}{32}\boldsymbol{b}_2]\right]$
Case 3 $(i,j) = (3,1)$	$[0.0498\boldsymbol{b}_1 - 0.0315\boldsymbol{b}_2, -0.0996\boldsymbol{b}_1 + 0.063\boldsymbol{b}_2, 0.0498\boldsymbol{b}_1 - 0.0315\boldsymbol{b}_2]$	$\left[\frac{1}{20}\boldsymbol{b}_1 - \frac{1}{32}\boldsymbol{b}_2, -\frac{2}{20}\boldsymbol{b}_1 + \frac{2}{32}\boldsymbol{b}_2, \frac{1}{20}\boldsymbol{b}_1 - \frac{1}{32}\boldsymbol{b}_2\right]$
Case 4 $(i,j) = (3,2)$	$[0.0498\boldsymbol{b}_1 - 0.0315\boldsymbol{b}_2, -0.0813\boldsymbol{b}_1 + 0.0813\boldsymbol{b}_2, 0.0315\boldsymbol{b}_1 - 0.0498\boldsymbol{b}_2]$	$\left[\frac{1}{20}\boldsymbol{b}_1 - \frac{1}{32}\boldsymbol{b}_2, -\frac{2}{20}\boldsymbol{b}_1 + \frac{3}{32}\boldsymbol{b}_2, \frac{1}{20}\boldsymbol{b}_1 - \frac{2}{32}\boldsymbol{b}_2\right]$

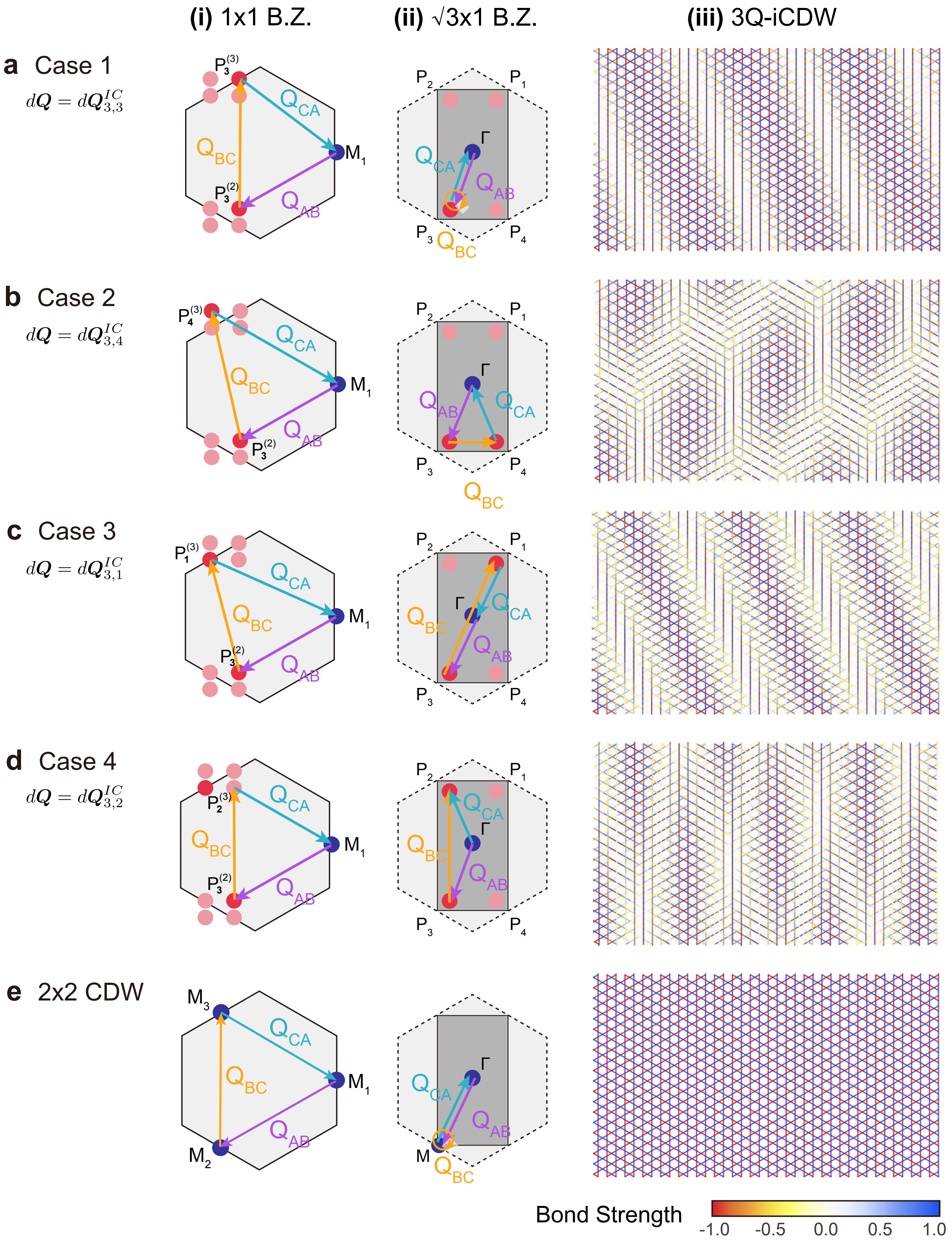

FIG. S22: **Schematic illustrations of incommensurate CDW (IC-CDW) order parameters.** (i) and (ii)-the columns show the wave-vectors of the CDW orders in the 1×1 and $\sqrt{3}\times1$ Brillouin zones (BZs), respectively. (iii)-th column delineates the corresponding 3Q bond modulations in real space to exemplify the case. **(a-d)** IC-CDW orders. **(e)** Commensurate 2×2 CDW order. The type-I (type-II) VHSs are marked by blue (red) circles in the 1×1 and $\sqrt{3}\times1$ BZs (i,ii of **a-e**). The order parameters are constructed, such that one of the three Q vectors,Q_{BC} ,connects two (equivalent or inequivalent) type-II P_i out of four (P_1, P_2, P_3, and P_4) and the rest two Q vectors,(Q_{AB}, Q_{AC}), connect the chosen P_i and the VHS at Γ, respectively. This construction results in four symmetry-inequivalent order parameters listed in **a-d**. The shifted bond Q vectors $d\boldsymbol{Q}$ are chosen as $d\boldsymbol{Q}^{IC}_{3,3}$,$d\boldsymbol{Q}^{IC}_{3,4}$,$d\boldsymbol{Q}^{IC}_{3,1}$,and $d\boldsymbol{Q}^{IC}_{3,2}$ (i,ii of **a-d**). The 2×2 CDW corresponds to $d\boldsymbol{Q}=0$ (i,ii of **e**).

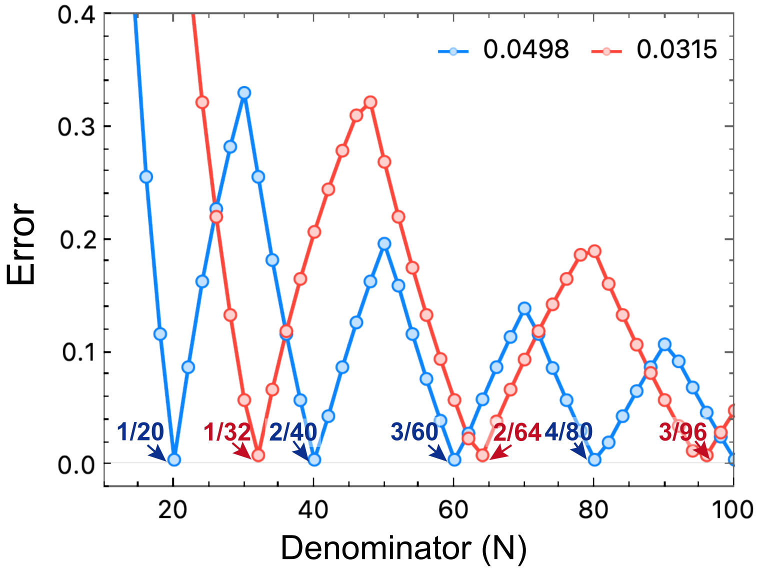

FIG. S23: **The error of commensurate approximation depending on the supercell size $N_1, N_2 < 100$.** To employ commensurate approximations, we approximate the two irrational values $(0.0498, 0.0315)$ to the closest rational numbers, expressed as a fraction n/N of two integers (n, N). Here, the numbers are inherited from $d\boldsymbol{q}_i$ (See Eqs.32,33), and the error is defined as $|\text{Exact} - \text{Approximated value}|/\text{Exact value}$. The approximated values $(1/20, 1/32)$ give the local minimum in the error with the actual values $(0.0498, 0.0315)$, thus we perform 20×32 supercell calculations.

Supplementary Note 12. Engineering of VHS

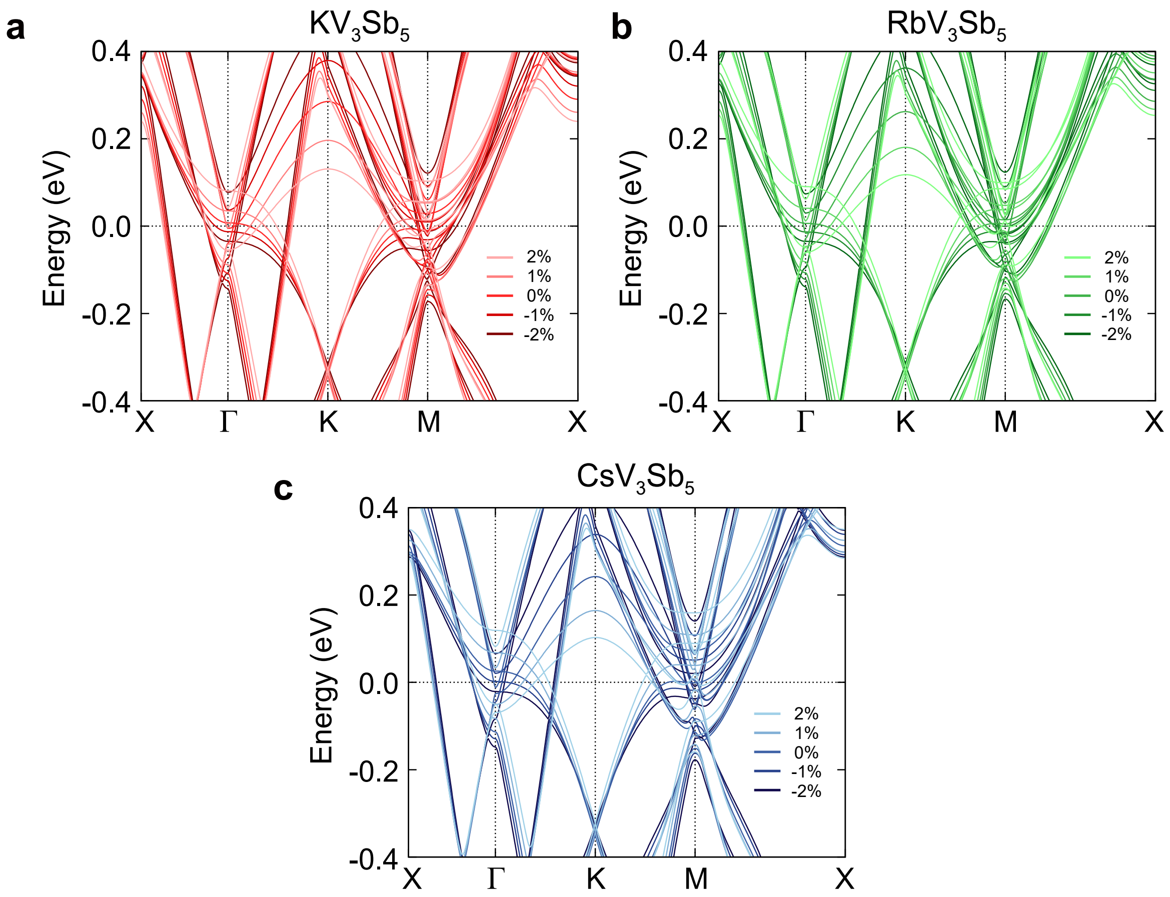

FIG. S24: **Strain engineering of VHSs in the AV_3Sb_5 monolayer. a-c** DFT Band structures of the AV_3Sb_5 monolayer family as a function of biaxial strain.

Figures S24**a-c** show DFT band structures as a function of biaxial strain for the AV_3Sb_5 monolayer family. Under the strain, we find that saddle point bands near Γ and M points drastically shift in energy compared with other bands for all three alkali atoms. The tunability of VHS energy is captured in the variations of chemical potential μ in the TB model under the strain, as displayed in Fig. 5**c** in the main text. It shows that small strain magnitudes of $\sim \pm 2\%$ can lead to large changes of μ ranging from about 50 meV to about -100 meV.

We also examine the doping effect on the location of VHSs. We simulate the doping effect by changing the number of alkali atom A as $A_{1+x}V_3Sb_5$ using the $2\sqrt{3} \times 2$ supercell: $x > 0$ ($x < 0$) corresponds to the electron (hole) doping. Figures S25**a-c** show the unfolded band structures obtained at $x = 0.5$ and -0.5 for all three alkali atoms. The doping effect

leads to the tuning of locations of VHSs, which is captured in the variation of chemical potential μ of the TB model in Fig. 5**d** in the main text.

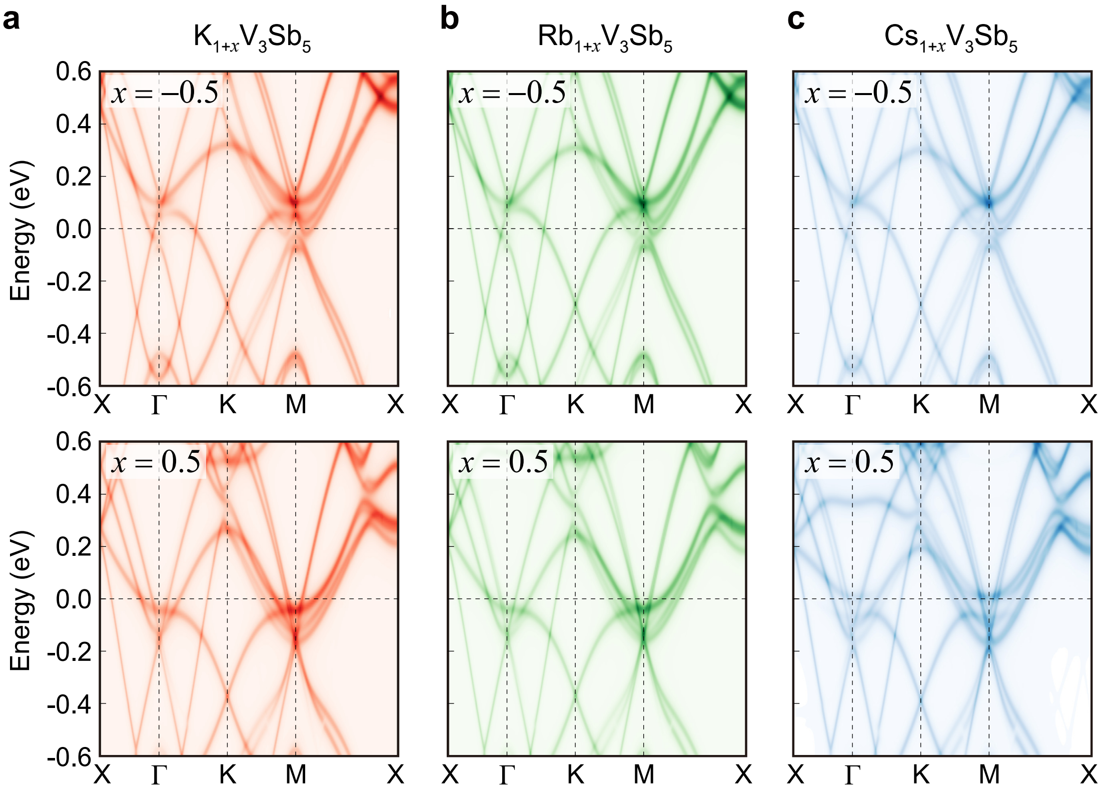

FIG. S25: **Doping engineering of VHSs in the AV_3Sb_5 monolayer. a-c** DFT band structures of the $A_{1+x}V_3Sb_5$ monolayer family at doping level $x = -0.5$ and 0.5. Here, we use the $2\sqrt{3} \times 2$ supercell to simulate the doping effect and the band structures are unfolded into BZ of the $\sqrt{3} \times 1$ unit cell by using the method proposed by Popescu and Zunger [25] as implemented in the VaspBandUnfolding code [26].

Supplementary References

[1] Zhao, H. *et al.* Cascade of correlated electron states in the kagome superconductor CsV_3Sb_5. *Nature* **599**, 216–221 (2021).

[2] Chen, H. *et al.* Roton pair density wave in a strong-coupling kagome superconductor. *Nature* **599**, 222–228 (2021).

[3] Liang, Z. *et al.* Three-dimensional charge density wave and surface-dependent vortex-core states in a kagome superconductor CsV_3Sb_5. *Phys. Rev. X* **11**, 031026 (2021).

[4] Xu, H.-S. *et al.* Multiband superconductivity with sign-preserving order parameter in kagome superconductor CsV_3Sb_5. *Phys. Rev. Lett.* **127**, 187004 (2021).

[5] Yu, J. *et al.* Evolution of electronic structure in pristine and Rb-reconstructed surfaces of kagome metal RbV_3Sb_5. *Nano Lett.* **22**, 918–925 (2022).

[6] Tan, H., Liu, Y., Wang, Z. & Yan, B. Charge density waves and electronic properties of superconducting kagome metals. *Phys. Rev. Lett.* **127**, 046401 (2021).

[7] Wu, X. *et al.* Nature of unconventional pairing in the kagome superconductors AV_3Sb_5 ($A =$ K, Rb, Cs). *Phys. Rev. Lett.* **127**, 177001 (2021).

[8] Zhou, S. & Wang, Z. Chern fermi-pockets and chiral topological pair density waves in kagome superconductors. *arXiv:2110.06266 [cond-mat]* (2021).

[9] LaBollita, H. & Botana, A. S. Tuning the van hove singularities in AV_3Sb_5 ($A =$ K, Rb, Cs) via pressure and doping. *Phys. Rev. B* **104**, 205129 (2021).

[10] Altland, A. & Simons, B. D. *Condensed matter field theory* (Cambridge university press, 2010).

[11] Jiang, Y.-X. *et al.* Unconventional chiral charge order in kagome superconductor KV_3Sb_5. *Nat. Mater.* **20**, 1353–1357 (2021).

[12] Mielke, C. *et al.* Time-reversal symmetry-breaking charge order in a kagome superconductor. *Nature* **602**, 245–250 (2022).

[13] Wang, Z. *et al.* Electronic nature of chiral charge order in the kagome superconductor CsV_3Sb_5. *Phys. Rev. B* **104**, 075148 (2021).

[14] Shumiya, N. *et al.* Intrinsic nature of chiral charge order in the kagome superconductor RbV_3Sb_5. *Phys. Rev. B* **104**, 035131 (2021).

[15] Aryasetiawan, F. *et al.* Frequency-dependent local interactions and low-energy effective models from electronic structure calculations. *Phys. Rev. B* **70**, 195104 (2004).

[16] Jeong, M. Y. *et al.* Crucial role of out-of-plane Sb p orbitals in van hove singularity formation and electronic correlations in the superconducting kagome metal CsV_3Sb_5. *Phys. Rev. B* **105**, 235145 (2022).

[17] Şaşıoğlu, E., Friedrich, C. & Blügel, S. Effective coulomb interaction in transition metals from constrained random-phase approximation. *Phys. Rev. B* **83**, 121101 (2011).

[18] Waldecker, L. *et al.* Rigid band shifts in two-dimensional semiconductors through external dielectric screening. *Phys. Rev. Lett.* **123**, 206403 (2019).

[19] Kim, M. *et al.* Control of electron-electron interaction in graphene by proximity screening. *Nature communications* **11**, 1–6 (2020).

[20] Roy, B. & Foster, M. S. Quantum multicriticality near the dirac-semimetal to band-insulator critical point in two dimensions: A controlled ascent from one dimension. *Phys. Rev. X* **8**, 011049 (2018).

[21] Nie, L., Tarjus, G. & Kivelson, S. A. Quenched disorder and vestigial nematicity in the pseudogap regime of the cuprates. *Proceedings of the National Academy of Sciences* **111**, 7980–7985 (2014).

[22] Yu, G., Wu, Z., Zhan, Z., Katsnelson, M. I. & Yuan, S. Dodecagonal bilayer graphene quasicrystal and its approximants. *npj Computational Materials* **5**, 1–10 (2019).

[23] Troyer, M. & Zürich, E. Classical and quantum monte carlo algorithms and exact diagonalization (2004).

[24] Kautzsch, L. *et al.* Incommensurate charge-stripe correlations in the kagome superconductor $CsV_3Sb_{5-x}Sn_x$. *arXiv:2207.10608 [cond-mat]* (2022).

[25] Popescu, V. & Zunger, A. Extracting E versus k effective band structure from supercell calculations on alloys and impurities. *Phys. Rev. B* **85**, 085201 (2012).

[26] Zheng, Q. VaspBandUnfolding. https://github.com/QijingZheng/VaspBandUnfolding.
